# YAP 5-methylcytosine modification increases its mRNA stability and promotes the transcription of exosome secretion-related genes in lung adenocarcinoma

**DOI:** 10.1038/s41417-022-00533-7

**Published:** 2022-09-19

**Authors:** Wenjun Yu, Congcong Zhang, Yikun Wang, Xiaoting Tian, Yayou Miao, Fanyu Meng, Lifang Ma, Xiao Zhang, Jinjing Xia

**Affiliations:** 1 Department of Clinical Laboratory Medicine, Shanghai Chest Hospital, Shanghai Jiao Tong University School of Medicine, 200030 Shanghai, China; 2 Anhui University of Science and Technology School of Medicine, Huainan, 232001 Anhui China; 3 Shanghai Institute of Thoracic Oncology, Shanghai Chest Hospital, Shanghai Jiao Tong University School of Medicine, 200030 Shanghai, China; 4 Department of Pulmonary Medicine, Shanghai Chest Hospital, Shanghai Jiao Tong University School of Medicine, 200030 Shanghai, China

**Keywords:** Cell biology, Non-small-cell lung cancer

## Abstract

YAP is a transcriptional co-activator with critical roles in tumorigenesis. However, its upstream regulatory mechanism, especially how its mRNA stability is regulated, remains to be further studied. Here, we validated that YAP expression was higher in lung adenocarcinoma (LUAD) tissues compared to adjacent normal tissues, and found that YAP m^5^C modification occurred in its 328–331 3′ UTR region under the promotion NSUN2 and ALYREF, and increased the stability of YAP mRNA. This m^5^C modification also inhibited miR-582-3p binding and m^6^A modification in the nearby region. In addition, YAP m^5^C modification enhanced the exosome secretion effect, which was caused by two YAP-dependent transcription factors, Mycn and SOX10, and then stimulating the transcription of seven downstream exosome-promoting genes. Furthermore, we found that YAP m^5^C modification and its exosome-secretion-promoting function contributed to the malignant phenotype and AZD9291 (a third-generation EGFR-TKI) resistance of LUAD cells. Collectively, YAP is promoted by its m^5^C modification, and blocking YAP m^5^C modification will be helpful for future LUAD treatment.

## Abstract (web summary)

Briefly, YAP 3′UTR is m^5^C modified under the promotion of NSUN2 and ALYREF, and this modification increases YAP mRNA stability and inhibits nearby m^6^A modification and miR-582-3p binding. Furthermore, YAP stimulates seven exosome biogenesis, transport and release-related genes including *HRS*, *PLD2*, *RAB2B*, *RAB27A*, *RAB27B*, *VAMP* and *ATG7* in a Mycn- and SOX10-dependent manner.

## Graphical abstract

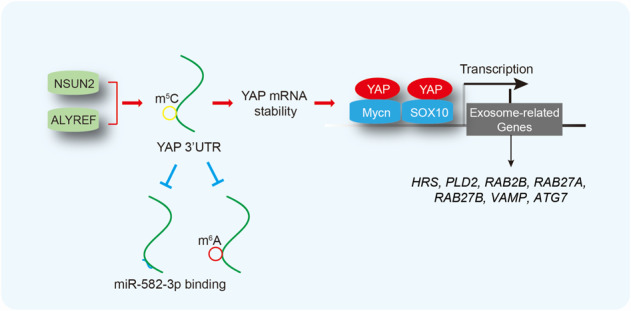

## Introduction

YAP is a cancer-promoting transcription co-factor downstream of Hippo pathway [1]. Transcription factors related to YAP include TEAD family, SMAD family, RUNX family, etc [2, 3]. Our Lab found that transcription factor TFCP2 regulated by YAP to promote the transcription of ferritin [4, 5]. Additionally, TFCP2 also recruits transcription factors for YAP, including FOXC1, FOXA1, etc., to promote cancer [6]. We also found that transcription-promoting activity of YAP is controlled by post-translational modifications such as O-GlcNAcylation, which can inhibit YAP phosphorylation [7]. In lung adenocarcinoma (LUAD), YAP can promote tumor cell proliferation and stemness [8]. High expression of YAP predicts poor prognosis in EGFR-mutant patients [9]. YAP 331 arginine to tryptophan (R331W) mutation is an allele predisposed for LUAD with high familial penetrance [10]. The above findings suggest that YAP is a promoting factor for LUAD. However, the mechanism by which YAP activates and promotes LUAD is still not fully understood.

The main function of YAP is to control organ size by regulating cell proliferation and contact inhibition [11]. In tumors, high expression of YAP causes cells to acquire stemness, and echoes the effect of epithelial-to-mesenchymal transition (EMT) [12]. Therefore, tumors with high expression of YAP have a strong ability of invasion and metastasis [13]. Under hypoxia, YAP can bind and maintain the stability of HIF1α, and further promote the transcription of PKM2 gene to stimulate glycolysis [14]. Our Lab elucidated that YAP promotes NUDT9 and SLC5A3 transcription and activates the hexosamine biosynthesis pathway (HBP) [7]. YAP also promotes tumor progression by regulating tumor microenvironment, for example by promoting transcription of CCL2, a macrophage-specific chemokine, to recruit tumor-associated macrophages [15]. In tumor microenvironment, exosome is a type of extracellular vesicle that originated from endocytosis and contains a broad repertoire of cargoes, including nucleic acids (i.e., DNA, mRNA, microRNA, long and short noncoding RNA) and proteins (i.e., cytoskeletal proteins, transmembrane proteins, heat shock proteins and ATPase) [16, 17]. As a master regulator of cellular signaling, exosome orchestrates various autocrine and paracrine functions to alter tumor microenvironment, growth and progression [17]. Tumor cells can transfer malignant phenotype to normal cells via exosome, and establish a fertile local and distant microenvironment to help cancer cell growth [18]. However, studies on the role of YAP in promoting exosomes are limited. YAP is also an important factor promoting drug resistance in cancer [19]. Targeted therapy for LUAD has achieved promising effects in the treatment of some driver gene mutations such as EGFR mutation [20]. Currently, third-generation EGFR-tyrosine kinase inhibitor (TKI) has been used in first-line treatment for LUAD, which can target T790M mutation resistance caused by first generation EGFR-TKI, and other EGFR oncogenic mutations such as exon 19 deletion [21, 22]. However, third-generation EGFR-TKI further generates new drug resistance such as C797S mutation [23]. Therefore, whether other proto-factors such as YAP can lead to third-generation EGFR-TKI resistance remains to be studied.

YAP can be phosphorylated by the core kinase in Hippo pathway, LATS1/2, to induce cytoplasmic localization, and subsequent ubiquitin mediated degradation driven by the E3-ligase βTrCP [24]. However, some regulation patterns to YAP are independent of Hippo pathway. For example, our Lab also revealed that YAP can be O-GlcNAcylated at Thr241 and this O-GlcNAcylation event antagonizes Hippo pathway-mediated phosphorylation of YAP [7]. 3′ untranslated region (3′UTR) is an essential region in mRNA and have important functions in regulating mRNA localization, mRNA stability, and translation [25]. Most functions of 3′ UTRs are mediated by RNA-binding proteins [25]. For example, YAP 3′UTR can be alternative spliced in a splicing factor hhnRNP F-dependent manner, and further lead to entire mRNA degradation [26]. However, microRNA binding and RNA modification (such as N^6^-methyladenosine (m^6^A) modification, the most abundant internal modification of RNA in eukaryotic cells which occurs at the consensus motif RRACH (R = A or G, H = A, C or U) [27]) also play independent regulatory roles in 3′UTR [28]. For instance, miR-195-5p and miR-381 are two microRNAs that are identified to directly bind to YAP 3′UTR to induce YAP mRNA degradation [29, 30]. Our Lab also found that both miR-7 and miR-582-3p binds to YAP 3′ UTR to result in degradation of YAP mRNA, while m^6^A modification near the binding site of miR-582-3p on YAP 3′ UTR can inhibit the miR-582-3p binding [31, 32]. 5-methylcytosine (m^5^C) modification, another type of abundant RNA methylation, is catalyzed by 5-methylcytosine RNA methyltransferase to transfer a methyl group to the fifth carbon of a cytosine base in RNA sequences [33]. m^5^C has been frequently associated with tRNA, but also found in mRNAs most often in their 5′ and 3′ UTR regions [34], and has emerged as an important regulator of many aspects of gene expression, including RNA export, ribosome assembly, translation, and RNA stability [35]. NSUN2, a major m^5^C mRNA methyltransferase, has been found overexpressed and activated in many types of cancer including LUAD [36]. However, how m^5^C modification and NSUN2 promote cancer, and how they function through YAP still remains largely unknown.

In this study, we elucidated YAP mRNA stability was increased by its m^5^C modification, which was NSUN2- and ALYREF-dependent. Moreover, m^5^C modification of YAP enhanced its promoting effect of exosome secretion. YAP m^5^C modification and its function to promote exosome secretion contributed to the transformation phenotype and AZD9291 resistance of LUAD cells. Targeting YAP m^5^C might be helpful for treating LUAD in the future.

## Materials and methods

### Tissue samples

All the tissue and plasma specimens (Shanghai, China, mean age ± SD, 60.05 ± 8.37 years; male: female ratio, 1.04:1) were recruited in Shanghai Chest Hospital (Shanghai, China) from March 2015 to December 2021. The diagnostic power was analyzed by receiver operating characteristic (ROC) curve analysis.

### Immunoblotting (IB) and enzyme linked immunosorbent assay (ELISA)

Protein levels were analyzed using IB and ELISA following conventional protocols. For IB, the primary antibodies used were anti-YAP (Abcam, Cambridge, MA, USA, #ab52771), anti-GAPDH (CST, Boston, MA, USA, #5174 and #51332), anti-p-127-YAP (Abcam, #ab76252), anti-p-381-YAP (CST, #13619), anti-O-Glc-YAP (developed by Biolynx, Hangzhou, China), anti-HRS (Abcam, #ab72053), anti-PLD2 (CST, #13904), anti-RAB2B (Novus, Littleton, CO, USA, #NBP1-31631), anti-RAB27A (Abcam, #ab55667), anti-RAB27B (Abcam, #ab103418), anti-VAMP (Abcam, #ab36195), anti-ATG7 (Abcam, #ab52472), anti-SOX10 (Abcam, #ab227680), anti-Mycn (Abcam, #ab227822), anti-ALYREF (Abcam, #ab202894), anti-NSUN2 (Abcam, #ab259941), anti-DNMT2 (Abcam, #ab220175), anti-NSUN1 (Abcam, #ab270175), anti-NSUN3 (Abcam, #ab272616), anti-NSUN4 (Abcam, #ab235430), anti-NSUN5 (Abcam, #ab121633), anti-NSUN6 (Novus, #NBP1-92202), anti-NSUN7 (Biorbyt, Cambridge, UK, #orb258175), anti-YBX1 (Abcam, #ab255606), anti-EXOSC10 (Abcam, #ab94981), anti-SPI1 (Abcam, #ab227835), anti-CD63 (Abcam, #ab271286), anti-TSG101 (Abcam, #12501), anti-ALIX (Abcam, #ab275377), anti-CD9 (Abcam, #ab92726), and anti-calnexin (Abcam, #ab133615). For ELISA, YAP and NSUN2 levels were measured using kits from Yingxin Biotech Ltd. (Shanghai, China) as per manufacturer’s instructions.

### Cell culture

Established A549, H1299, H1975, Wi-38, MRC-5 and BEAS-2B cell lines were purchased from Fuheng Biotechnology (Shanghai, China). H1975- and HCC827-based AZD9291 resistant cell lines were acquired from previous studies [5]. For 3D spheroid culture, basement membrane extract (BME) (Trevigen, Gaithersburg, MD, USA) was seeded in a 96-well plate at 50 μl/well and plated at 37 °C for 30 min. Then, cells with indicated genes overexpressed or knocked out were seeded on top of BME at a density of 1 × 10^5^ cells per well. After formation of spheroids, they were treated with AZD9291 (MedChemExpress, Monmouth, NJ, USA) with or without GW4869 (Sigma, St Louis, MO, USA) for 24 h. Images were captured after staining with SYTOX green (Invitrogen, Carbsland, CA, USA) [37].

### RNA-immunoprecipitation (RNA-IP)

For RIP assay, a Magna RIP Kit (Merck Millipore, Billerica, MA, USA) was used. Briefly, a total of 1 × 10^7^ cells were lysed in complete RNA lysis buffer, then cell lysates were incubated with RIP immunoprecipitation buffer containing magnetic beads conjugated with 5 μg anti-m^5^C (Abcam, #ab10805), anti-DNMT2 (Abcam, #ab220175), anti-NSUN1 (Abcam, #ab270175), anti-ALYREF (Abcam, #ab202894), anti-NSUN2 (Abcam, #ab259941), anti-DNMT2 (Abcam, #ab220175), anti-NSUN3 (Abcam, #ab272616), anti-NSUN4 (Abcam, #ab235430), anti-NSUN5 (Abcam, #ab121633), anti-NSUN6 (Novus, #NBP1-92202), anti-NSUN7 (Biorbyt, #orb258175), anti-YBX1 (Abcam, #ab255606) antibodies or control IgG (CST #3900) overnight at 4 °C. Next, the beads were washed with washing buffer and incubated with proteinase K at 55 °C for 30 min to remove the proteins. The remaining RNA was extracted using Trizol (Ambion) and reverse-transcribed into complementary DNA using the PrimeScript^TM^ RT reagent kit (Takara). The SYBR premix Ex Taq (Takara) kit was used for real-time qPCR [31].

### Regents and plasmids

For regents, ActD (MedChemExpress), CHX (Sigma), GW4869 (Sigma), AZD9291 (MedChemExpress). Plasmids DNMT2, NSUN1-7, ALYREF, YBX1, EXOSC10, Mycn, SOX10, SPI1, YAP-(-1k)-promoter, YAP-(-2k)-promoter, Mut4-, Mut5-, Mut6-YAP-3’ UTR overexpression plasmids were purchased from Biolink (Shanghai, China). miR-582-3p mimics, miR-582-3p inhibitors, WT-, Mut1-, Mut2- and Mut3-YAP-3′ UTR luciferase reporters, YAP knockout and YAP overexpression plasmids were acquired from previous studies [5, 7, 31]. Short hairpin RNAs (shRNAs) and lentiCRISPR v2-based constructs were used for knocking down and knocking out Mycn, SOX10, NSUN2 and ALYREF, respectively. The sequences for gRNAs and shRNAs were summarized in Table S1.

### Quantitative RT-PCR (qPCR)

Total RNA was extracted using Trizol (Ambion, Carlsbad, CA, USA) and reverse-transcribed into complementary DNA using the PrimeScript^TM^ RT reagent kit (Takara, Dalian, China). The SYBR premix Ex Taq (Takara) kit was used for real-time qPCR. The primers are listed in Table S1.

### Luciferase activity measurement

Luciferase activities were measured using a dual-luciferase kit (Promega, Madison, WI, USA) according to the manufacturer’s instructions. Firefly reporters (including WT-, Mut1- Mut2-, Mut3-, Mut4- Mut5- and Mut6-YAP-3′ UTR, as well as YAP-(-1k)-promoter, YAP-(-2k)-promoter luciferase reporters) were co-transfected into lung cancer cells with *Renilla* plasmids using Lipofectamine^™^ 2000 transfection reagent (Invitrogen). After incubation for 48 h, the cells were harvested and then lysed in the passive lysis buffer from the kit. The fluorescence intensity of luciferase reporters was then examined and normalized to the *Renilla* luciferase activity.

### Co-immunoprecipitation (co-IP)

co-IP was performed as described previously. Cell lysates were mixed with 50 ul protein A/G-magnetic beads (Novex, Oslo, Norway) and incubated at 4 °C overnight with the indicated antibodies. The beads were washed with Western/IP lysis buffer (Beyotime, Haimen, China), suspended in SDS-PAGE loading buffer and then detected by IB. The antibodies used for co-IP were: anti-YAP (Santa Cruz Biotechnology, Santa Cruz, CA, USA, #sc-101199).

### RNA probe precipitation

Biotin-labeled probe was synthesized by Sangon Biotech (Shanghai, China). Cells were fixed using 4% formaldehyde, then lysed, sonicated, and centrifuged. Next, the supernatant was extracted as input, and the remaining amount was incubated with indicated probe and Dynabeads M-280 Streptavidin (Thermo Scientific, Waltham, MA, USA) overnight at room temperature. The next day, the mixture was washed and incubated with lysis buffer and proteinase K to reverse the cross-linking. Finally, the RNA mixture was extracted using Trizol and was detected via qPCR [5–7, 31]. Probe sequence was listed in Table S1.

### Isolation and measurement of exosome

Exosome was isolated from cultured media of cells or plasma of human via three sequential centrifugation steps at 4 °C, (1) 15 min at 500 *g* to remove cells; (2) 30 min at 10,000 *g* to remove cell debris; and (3) ultracentrifugation at 110,000 *g* for 70 min to pellet exosome. The pellet was finally resuspended in PBS and centrifuged at 110,000 *g* for another 70 min to remove soluble and secreted proteins. The concentration and size of exosome were analyzed using nanoparticle tracking analysis (NTA) by a Nanosight NS 300 system (NanoSight Technology, Malvern, UK) [37].

### Immunofluorescence (IF), immunohistochemistry (IHC),

IF and IHC were performed according to the conventional protocols. For IF, PKH67 (Sigma, MKCG5294) was probed for marking exosome packaged in H1299 cells. For IHC, the primary antibodies used in the study were anti-YAP (Abcam, #ab52771).

### TEM analysis

Exosome was resuspended in 4% paraformaldehyde, and images were captured using the JEM1230 TEM (JEOL, Tokyo, Japan).

### Measurements of cell viability, caspase 3/7 activity and anchorage-independent colony formation

Cell viability was detected using a CCK8 kit (Beyotime). Caspase3/7 activity was measured using Caspase 3/7 Glo luciferase reagent (Promega). As for anchorage-independent colony formation assay, LUAD cells were seeded in a six-well plate containing 0.3% agarose at a density of 6 × 10^3^ cells per well. Two weeks later, the numbers of colonies were calculated under microscope.

### Mouse experiments

A549 cells with indicated genes overexpressed or knocked out (initial 5 × 10^6^) were subcutaneously injected into 6-week-old athymic nude mice (Jiesijie, Shanghai, China) with or without GW4869 treatment to acquire cell-derived xenograft mouse models. Fresh tissues in a size of 2 mm^3^ were subcutaneously implanted into six-week-old athymic nude mice to generate PDX mouse models. After successful passage, the PDX model could be used for further analysis. Tumor volumes were calculated as 0.5 × L × W^2^ (L indicating length while W indicating width) [5]. Histological sections were prepared using a microtome and subsequently stained with IHC or hematoxylin and eosin.

### Statistical analysis

Tests used in this study included student’s *t* test, one-way, two-way ANOVA, *χ*^2^ test, and the Spearman rank-correlation analysis. A *p* < 0.05 was considered statistically significant.

## Results

### YAP was highly expressed in LUAD

**Fig. 1:**
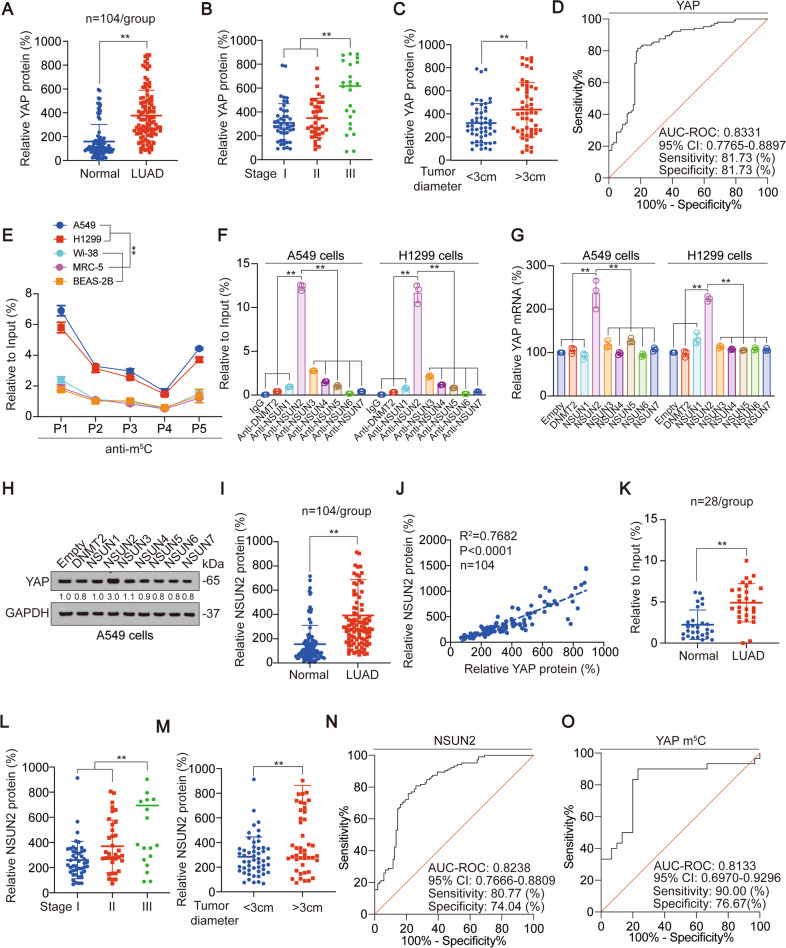
YAP m^5^C modification depends on NSUN2. **A** YAP protein levels were measured by ELISA in LUAD and adjacent normal tissues. **B**, **C** YAP protein levels in different stage (**B**) and tumor diameter (**C**) LUAD tissues. **D** ROC curves of YAP for discriminating LUAD from non-tumor tissues. **E** The enrichment of m^5^C in A549, H1299, Wi-38, MRC-5 and BEAS-2B cells at indicated regions (P1: 1–700, P2: 701–1400, P3: 1401–2100, P4: 2101–2800 P5: 2801–3493) of YAP 3′ UTR was calculated as the percentage of the input RNA via RNA-IP using the anti-m^5^C antibodies. **F** The enrichment of indicated m^5^C writers in A549 and H1299 cells at P1 region of YAP 3′ UTR was calculated as the percentage of the input RNA via RNA-IP using the indicated antibodies. **G** YAP mRNA level was measured by qPCR in A549 and H1299 cells with indicated plasmids overexpressed. **H** YAP protein level was measured by IB in A549 cells with indicated plasmids overexpressed. The level of proteins was normalized to that of GAPDH, and the normalized level of proteins in negative control groups were arbitrarily set to 1. **I** NSUN2 protein levels were measured in LUAD and adjacent normal tissues. **J** Correlation between plasma exosome concentration and tissue YAP protein level in LUAD samples. **K** YAP m^5^C levels were measured in LUAD and adjacent normal tissues. **L**, **M** NSUN2 protein levels in different stage (**L**) and tumor diameter (**M**) LUAD tissues. **N**, **O** ROC curves of NSUN2 and YAP m^5^C for discriminating LUAD from non-tumor tissues. The data are shown as the means ± SD from three independent experiments. Images of IB are representative ones of three independent experiments. ^**^p < 0.01 indicate statistical significance. The data from (**A**, **C**, **I**, **K**, **M**) were analyzed by a student’s *t* test. The data from (**B**, **F**, **G**, **L**) were analyzed by a one-way ANOVA test. The data from (**E**) were analyzed by a two-way ANOVA test. The data from (**J**) were analyzed by a Spearman rank-correlation analysis.

Firstly, we evaluate YAP expression in LUAD. After analyzed paired of LUAD and adjacent normal tissues, we found that YAP protein level was increased in LUAD samples (Fig. 1A). Also, YAP protein level was upregulated in stage III LUAD tissues compared to stage I and II LUAD tissues, as well as in >3 cm diameter LUAD tissues compared to <3 cm LUAD tissues (Fig. 1B, C). The diagnostic power was calculated by ROC curve, and the AUC-ROC of YAP was 0.8331, 95% CI was 0.7765–0.8897, the best sensitivity was 81.73%, and the best specificity was 81.73% (Fig. 1D). We also found YAP expression was not associated with patient age, gender and smoking habit (Fig. S1A). These data suggested YAP was highly expressed in LUAD.

### m^5^C modification of YAP depends on NSUN2

In our previous research, we found that RNA m^6^A modification exists at YAP 3′ UTR region [31]. We further explored whether m^5^C modification, another common RNA modification, exists at YAP 3′ UTR region. We divided YAP 3′ UTR region into 5 segments, and then found that m^5^C modification existed in all 5 segments, and the m^5^C modification abundance of P1 region (1–700) was the highest (Fig. 1E, Fig. S1B) in A549 and H1299 cells. We also tested YAP m^5^C modification in lung fibroblast cell lines Wi-38 and MRC-5, and bronchial epithelial cell line BEAS-2B, and found that m^5^C modification levels in Wi-38, MRC-5 and BEAS-2B were significantly lower than those in A549 and H1299 cells (Fig. 1E) Through the screening of m^5^C methyltransferase (including DNMT2, NSUN1-7 [35]), we found that NSUN2 bound to the P1 region more significantly (Fig. 1F, Fig. S1C). Moreover, NSUN2 increased YAP mRNA and protein expression, whereas other m^5^C methyltransferases did not have similar capabilities (Fig. 1G, H and Fig. S1D). In addition, NSUN2 protein level was increased in LUAD samples compared to adjacent normal tissues (Fig. 1I) and positively associated with YAP protein level (Fig. 1J). Also, increasing YAP m^5^C modification were found in LUAD samples (Fig. 1K). Like YAP, NSUN2 protein levels were upregulated in stage III LUAD tissues (Fig. 1L), as well as in >3 cm diameter LUAD tissues (Fig. 1M). The AUC-ROC of NSUN2 was 0.8238, 95% CI was 0.7666–0.8809, the best sensitivity was 80.77%, and the best specificity was 74.04% (Fig. 1N). The AUC-ROC of YAP m^5^C was 0.8133, 95% CI was 0.6970–0.9296, the best sensitivity was 90.00%, and the best specificity was 76.67% (Fig. 1O). These data implied that YAP m^5^C depends on NSUN2.

### m^5^C modification of YAP relies on ALYREF and increases YAP mRNA stability

**Fig. 2:**
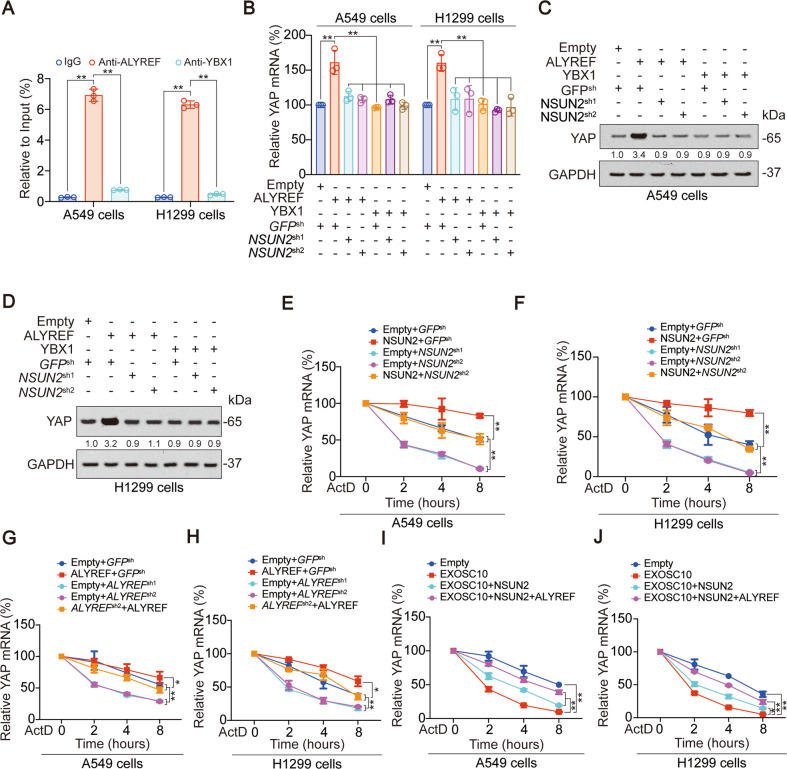
YAP m^5^C modification also relies on ALYREF and increases YAP mRNA stability. **A** The enrichment of ALYREF and YBX1 in A549 and H1299 cells at P1 region of YAP 3′ UTR was calculated as the percentage of the input RNA via RNA-IP using the anti-ALYREF and anti-YBX1 antibodies. **B** YAP mRNA level was measured by qPCR in A549 and H1299 cells with ALYREF or YBX1 overexpressed with or without NSUN2 knockdown. **C**–**D** YAP protein level was measured by IB in A549 (**C**) and H1299 (**D**) cells with ALYREF or YBX1 overexpressed with or without NSUN2 knockdown. The level of proteins was normalized to that of GAPDH, and the normalized level of proteins in negative control groups were arbitrarily set to 1. **E**–**J** YAP mRNA stability was measured in A549 and H1299 cells with NUSN2 (**E**–**F**), ALYREF (**G**–**H**) and EXOSC10 (**I**–**J**) overexpressed or knocked down at indicated time after ActD treatment. The data are shown as the means ± SD from three independent experiments. Images of IB are representative ones of three independent experiments. ^*^p < 0.05, ^**^p < 0.01 indicate statistical significance. The data from (**A**–**B**) were analyzed by a one-way ANOVA test. The data from (**E**–**J**) were analyzed by a two-way ANOVA test.

We subsequently investigated the reader that is responsible for YAP m^5^C. We found ALYREF, but not another reader YBX1, bound to the P1 region (Fig. 2A, Fig. S2A). ALYREF increased YAP mRNA and protein expression, whereas this effect was abolished by NSUN2 knockdown; meanwhile, YBX1 had no effect on up-regulating YAP (Fig. 2B–D, Fig. S2B). NSUN2 and ALYREF overexpression both upregulated, while NSUN2 and ALYREF knockdown down-regulated the stability of YAP mRNA. The effects of *NSUN2*^*sh2*^ and *ALYREF*^*sh2*^ which are designed for the 3′ UTR region could be reversed by their overexpression vectors, indicating that shRNAs do not have off-target effects (Fig. 2E–H). Overexpression of RNA exonucleases EXOSC10 significantly down-regulated the mRNA stability of YAP, but this effect was reversed by overexpression of NSUN2 and ALYREF (Fig. 2I, J and Fig. S2C), suggesting that YAP m^5^C modification repressed its 3′-5′mRNA degradation. The above results prove that m^5^C modification can occur in the 3′ UTR region of YAP, which can increase the stability of YAP mRNA.

### m^5^C modification of YAP inhibits miR-582-3p binding

**Fig. 3:**
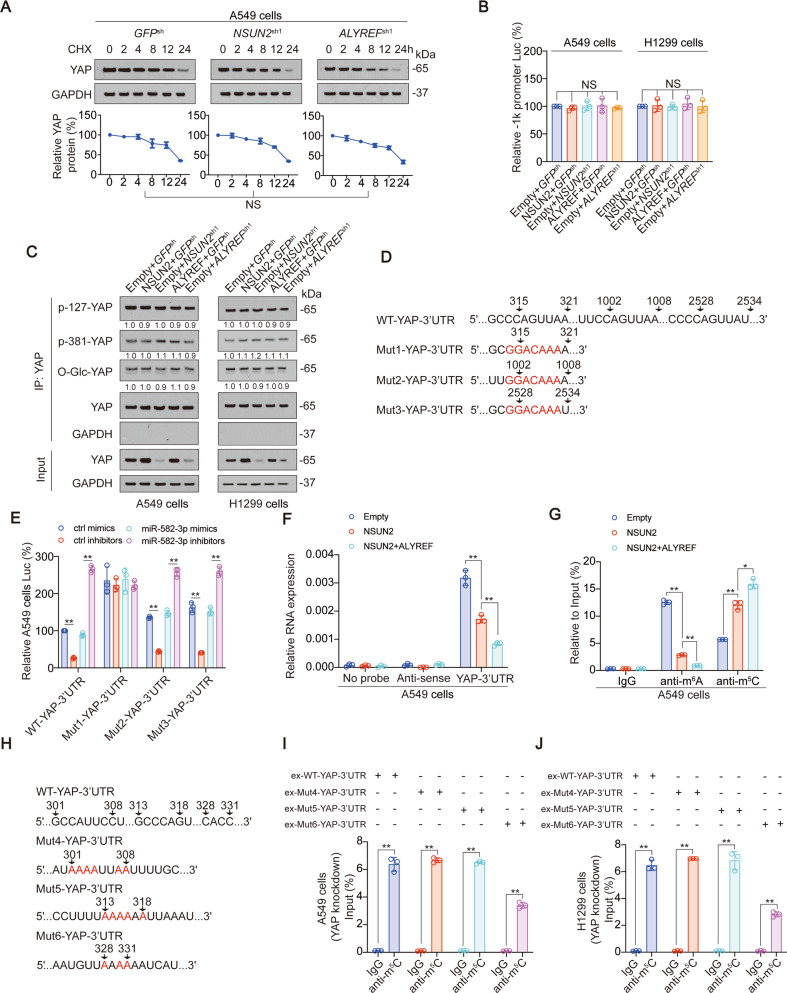
YAP m^5^C modification inhibits miR-582-3p interaction with YAP 3′ UTR. **A** YAP protein stability was measured in A549 cells with NSUN2 or ALYREF knockdown at indicated time after CHX treatment. **B** YAP -1k promoter activity was measured in A549 and H1299 cells with NUSN2 or ALYREF overexpressed or knocked down. **C** YAP phosphorylation and O-GlcNAcylation was measured in A549 and H1299 cells with NUSN2 or ALYREF overexpressed or knocked down. The level of proteins was normalized to that of co-immunoprecipitated YAP, and the normalized level of proteins in negative control groups were arbitrarily set to 1. The YAP level in each co-IP samples was adjusted to the same content. **D** WT and potential miR-582-3p binding motif mutation YAP 3′ UTR sequences were presented. Red fonts represented the mutant bases. **E** Activities of WT-YAP-3′ UTR, Mut1-YAP-3′ UTR, Mut2-YAP-3′ UTR, or Mut3-YAP-3′ UTR were measured in A549 cells with miR-582-3p overexpressed or knocked down. **F** miR-582-3p in NSUN2 with or without ALYREF overexpressed A549 cell lysis was pulled down and enriched with a YAP 3’ UTR P1 region probe and then detected by qPCR. **G** The enrichment of m^6^A and m^5^C in NSUN2 with or without ALYREF overexpressed A549 cells at P1 region of YAP 3′ UTR was calculated as the percentage of the input RNA via RNA-IP using the anti-m^6^A and anti-m^5^C antibodies. **H** WT and potential m^5^C modification motif mutation YAP 3′ UTR sequences were presented. Red fonts represented the mutant bases. **I**, **J** WT-YAP-3′ UTR, Mut4-YAP-3′ UTR, Mut5-YAP-3′ UTR, or Mut6-YAP-3′ UTR was overexpressed in YAP knockdown A549 (**I**) or H1299 (**J**) cells. The enrichment of m^5^C at P1 region of YAP 3′ UTR was calculated as the percentage of the input RNA via RNA-IP using the m^5^C antibodies. The data are shown as the means ± SD from three independent experiments. Images of IB are representative ones of three independent experiments. ^*^p < 0.05, ^**^p < 0.01 indicate statistical significance. NS, non-significant. The data from (**A**) were analyzed by a two-way ANOVA test. The data from (**B**, **E**–**G**) were analyzed by a one-way ANOVA test. The data from (**I**, **J**) were analyzed by a student’s *t* test.

We further explored the mechanism by which YAP m^5^C modification affects YAP expression. Firstly, we found that although NSUN2 and ALYREF knockdown significantly inhibited YAP m^5^C modification (Fig. S3A, B), they had no influence on the protein stability, promoter activity, phosphorylation and O-GlcNAcylation of YAP (Fig. 3A–C, Fig. S3C–D). In previous studies, we have found that miR-582-3p can bind to the 3′ UTR region of YAP to promote YAP mRNA degradation [31]. Here, we observed that when the predicted binding motif close to the 5′end (315–321 region in YAP 3′ UTR) was mutated (shown as Mut1-YAP-3′ UTR), the miR-582-3p mimics and miR-582-3p-inhibitors lost their abilities to regulate the activity of YAP 3′ UTR. However, miR-582-3p still significantly regulated the luciferase activities of the other two reporters with mutated 1002–1008 or 2528–2534 region in YAP 3′ UTR (two predicted miR-582-3p binding motifs in 3′ UTR), validating that the 315–321 region in YAP 3′ UTR might be the miR-582-3p binding motif in both liver cancer and LUAD cell lines (Fig. 3D, E and Fig. S3E) [31]. Overexpression of NSUN2 and ALYREF could significantly inhibit the binding of YAP 3′ UTR to miR-582-3p (Fig. 3F, Fig. S3F), enhance the m^5^C modification while inhibiting the m^6^A modification (which can promote the binding of YAP 3′ UTR and miR-582-3p [31]) (Fig. 3G, Fig. S3G). Since m^5^C modification occurs in GC-rich sequences [38–40], and m^5^C modification of YAP 3′ UTR P1 segment was closely related to miR-582-3p binding and m^6^A modification, we searched for three GC-rich motifs near the miR-582-3p binding m^6^A modification sites, and mutated G and C to A (Fig. 3H). Compared to WT-YAP-3′ UTR, Mut6-YAP 3′ UTR (328~331 region mutated) significantly inhibited YAP m^5^C modification, whereas Mut4 (301–308 region mutated) and Mut5 (313–318 region mutated) had no influence (Fig. 3I, J). The above data suggested that YAP m^5^C modification inhibited the binding between miR-582-3p and YAP 3′ UTR to prevent YAP degradation, and 328–331 motif might be the key motif of YAP m^5^C modification.

### YAP stimulates exosome secretion via Mycn- and SOX10-dependent transcription

**Fig. 4:**
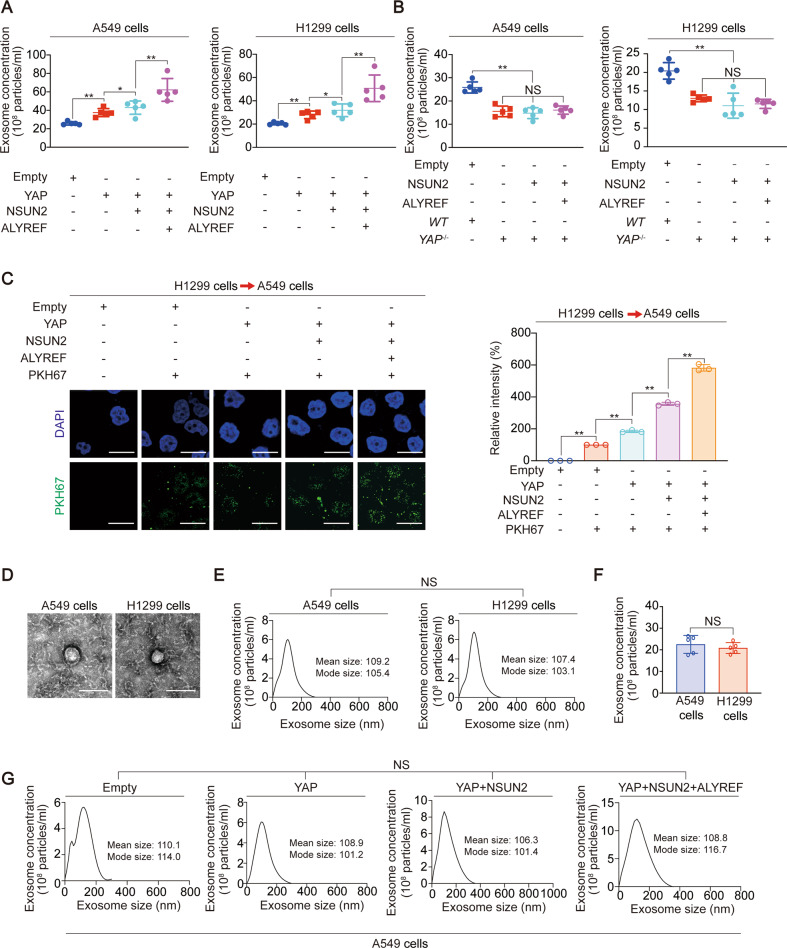
YAP m^5^C modification increases exosome concentration. **A**–**B** Exosome concentration was measured in A549 and H1299 cells with YAP overexpressed (**A**) or knocked out (**B**), with or without NSUN2 and ALYREF overexpressed. **C** The exosome from H1299 cells with YAP, NSUN2 with or without ALYREF overexpression was marked by PKH67 (green) and incubated with A549 cells. Afterwards, IF was performed for the detection of exosome. Scale bar, 50 µm. **D** Representative TEM images of plasma exosome in A549 and H1299 cells. Scale bar, 300 nm. **E** Relationship between exosome concentration and size in A549 and H1299 cells. The mean and mode size of exosome were shown. **F** Exosome concentration in A549 and H1299 cells. **G** Relationship between exosome concentration and size in A549 cells with YAP, NSUN2 with or without ALYREF overexpression. The mean and mode size of exosome were shown. The data are shown as the means ± SD from 3 to 5 independent experiments. ^*^p < 0.05, ^**^p < 0.01 indicate statistical significance. NS, non-significant. The data from (**A**–**C**) were analyzed by a one-way ANOVA test. The data from (**E**, **G**) were analyzed by a two-way ANOVA test. The data from (**F**) were analyzed by a student’s *t* test.

Whether YAP and its m^5^C modification regulate exosome? We found that YAP promoted exosome secretion of A549 and H1299 cells, and NSUN2 and ALYREF could further promote this ability (Fig. 4A). When YAP was knocked out, the exosome secretion of A549 and H1299 cells decreased, and this effect could not be reversed by NSUN2 and ALYREF, suggesting that the ability of NSUN2 and ALYREF to promote exosome secretion depends on YAP (Fig. 4B). PKH67 staining experiments further confirmed the above suggestions (Fig. 4C, Fig. S4A). We also found that YAP protein level was positively associated with exosome concentrations in paired of LUAD plasma and tissue samples (Fig. S4B). The exosomes were examined by transmission electron microscope (TEM) analysis (Fig. 4D). The exosome biomarkers including CD63, TSG101, ALIX and CD9 were detected, and endoplasmic reticulum (ER) biomarker calnexin was used as the negative control to validate the quality of the exosome (Fig. S4C). However, the size and concentration of exosomes in A549 and H1299 cells were close, and YAP, NSUN2 and ALYREF could not significantly change the size of exosomes (Fig. 4E–G).

**Fig. 5:**
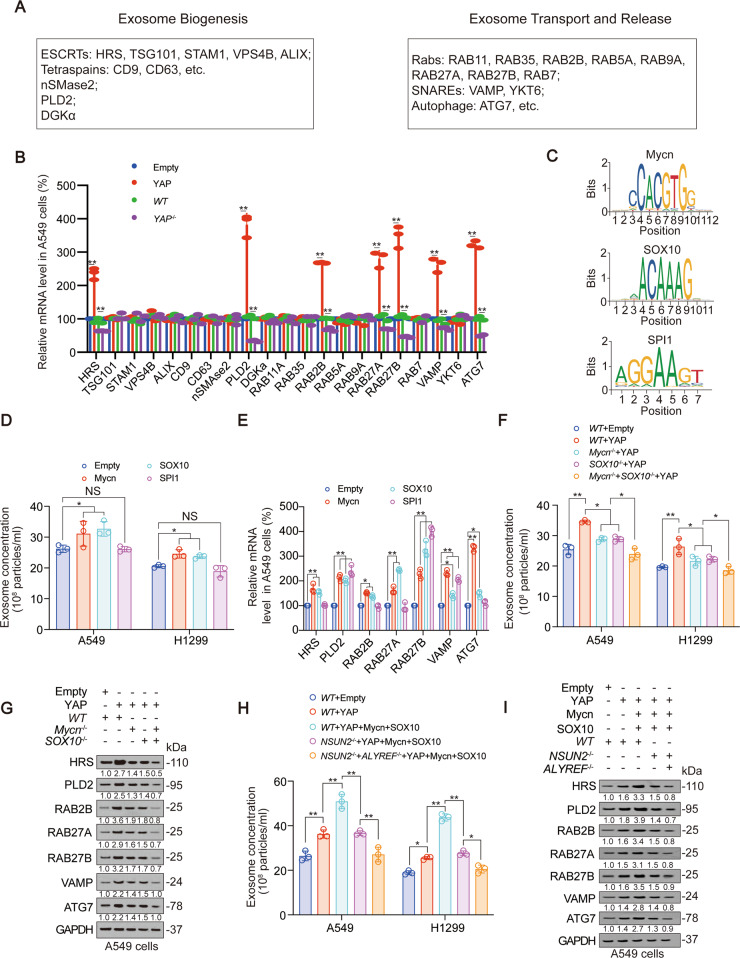
YAP stimulates transcription of exosome secretion genes via Mycn and SOX10. **A** Genes related to exosome biogenesis, transport and release were listed. **B** Indicated mRNA levels were measured by qPCR in YAP overexpressed or knocked out A549 cells. **C** Mycn, SOX10 and SPI1 binding motif found from JASPAR database. **D**, **E** Exosome concentration (**D**) and HRS, PLD2, RAB2B, RAB27A, RAB27B, VAMP and ATG7 mRNA levels (**E**) in A549 cells with Mycn, SOX10 or SPI1 overexpressed. **F**, **G** Exosome concentration (**F**) and HRS, PLD2, RAB2B, RAB27A, RAB27B, VAMP and ATG7 protein levels (**G**) in A549 cells with YAP overexpressed with or without Mycn or SOX10 knockout. The level of proteins was normalized to that of GAPDH, and the normalized level of proteins in negative control groups were arbitrarily set to 1. **H**, **I** Exosome concentration (**H**) and HRS, PLD2, RAB2B, RAB27A, RAB27B, VAMP and ATG7 protein levels (**I**) in A549 cells with indicated genes overexpressed or knocked out. The level of proteins was normalized to that of GAPDH, and the normalized level of proteins in negative control groups were arbitrarily set to 1. The data are shown as the means ± SD from three independent experiments. Images of IB are representative ones of three independent experiments. ^*^p < 0.05, ^**^p < 0.01 indicate statistical significance. The data from (**B**) were analyzed by a student’s *t* test. The data from (**D**–**F**, **H**) were analyzed by a one-way ANOVA test.

We further explored how YAP stimulates exosome secretion. We screened a series exosome biogenesis, transport and release-related genes to evaluate whether they were regulated by YAP [41–43]. We found YAP overexpression increased, while YAP knockout decreased mRNA level of *HRS*, *PLD2*, *RAB2B*, *RAB27A*, *RAB27B*, *VAMP* and *ATG7* (Fig. 5A, B and Fig. S5A). Using LASAGNA database, we found that there were binding motifs of Mycn, SOX10 and SPI1 in the promoter regions of the above 7 genes (Fig. 5C). Compared to SPI1, Mycn and SOX10 could significantly promote exosome secretion, and the mRNA levels of 7 exosome secreting genes (Fig. 5D, E, Fig. S5B). In addition, Mycn or SOX10 knockout partially reversed, while Mycn and SOX10 simultaneous knockout almost completely reversed the promotion effect of YAP on exosome secretion, as well as mRNA and protein expression of 7 exosomal secreting genes (Fig. 5F, G, Fig. S5C–E). Also, NSUN2 single knockout partially reversed, while NSUN2 and ALYREF simultaneous knockout almost completely reversed the promotion effect of YAP, SOX10 and Mycn (Fig. 5H, I, Fig. S5F–G). In all, these data revealed a YAP-mediated of SOX10 and Mycn exosome secretion-promoting transcription system.

### YAP m^5^C modification and downstream exosome secretion-promoting transcription system are essential for LUAD transformation phenotypes

**Fig. 6:**
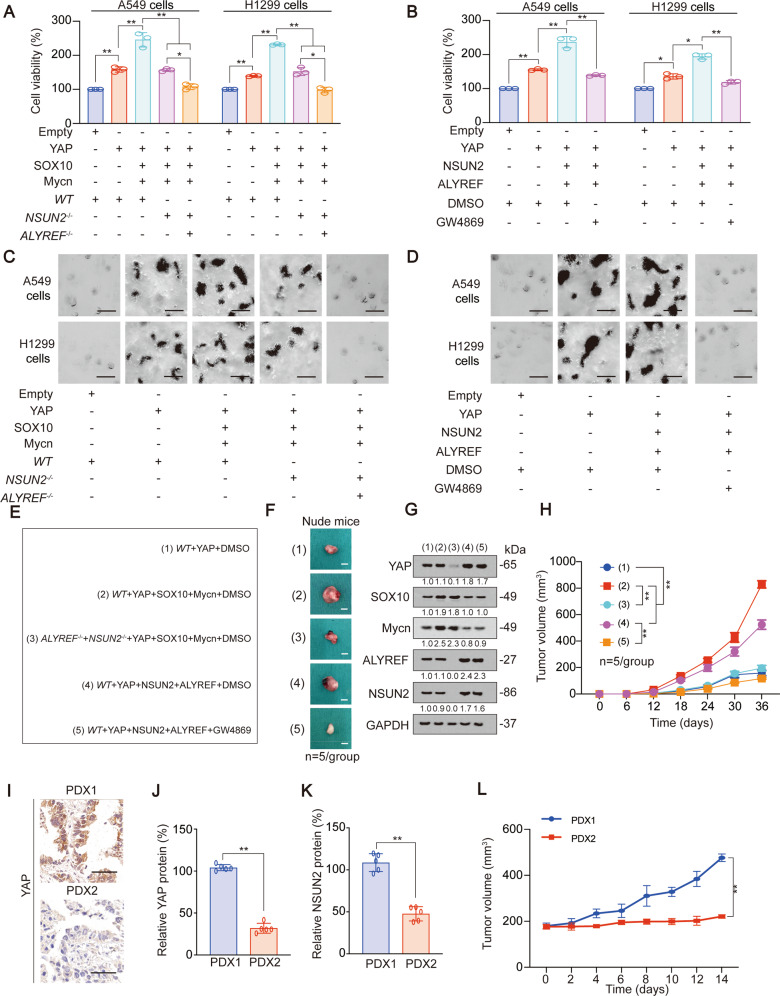
YAP m^5^C modification and downstream exosome secretion-promoting transcription system are essential for LUAD transformation phenotypes. **A**, **B** Cell viability was measured in A549 or H1299 cells with indicated genes overexpressed or knocked out. **C**, **D** Colony formation was measured in A549 or H1299 cells with indicated genes overexpressed or knocked out, and represented images were shown, scale bar, 100 µm. **E**–**H** Xenografts formed by A549 cells with indicated treatment (Group 1: YAP overexpression in *WT* cells with DMSO treatment. Group 2: YAP, SOX10 and Mycn overexpression in *WT* cells with DMSO treatment. Group 3: YAP, SOX10 and Mycn overexpression in *ALYREF*^*−/−*^ and *NSUN2*^*−/*−^ cells with DMSO treatment. Group 4: YAP, NSUN2 and ALYREF overexpression in *WT* cells with DMSO treatment. Group 5: YAP, NSUN2 and ALYREF overexpression in *WT* cells with GW4869 treatment (2 mg/kg, 3 weeks). Legends for each group (*n* = 5) were summarized in (**E**). Represented xenograft images were shown in (**F**). YAP, SOX10, Mycn, ALYREF and NSUN2 expressions were validated by IB (**G**). Tumor volume was monitored for 36 days (**H**). The level of proteins was normalized to that of GAPDH, and the normalized level of proteins in negative control groups were arbitrarily set to 1. **I** YAP expression in PDX1 and PDX2 tissues as measured by IHC, scale bar, 100 µm. **J**, **K** YAP (**J**) and NSUN2 (**K**) protein level in PDX1 and PDX2 tissues. **L** Tumor volume of PDX1 and PDX2 as monitored in 14 days. The data are shown as the means ± SD from 3 to 5 independent experiments. ^*^p < 0.05, ^**^p < 0.01 indicate statistical significance. The data from (**A**, **B**) were analyzed by a one-way ANOVA test. The data from (**H**, **L**) were analyzed by a two-way ANOVA test. The data from (**J**, **K**) were analyzed by a student’s *t* test.

Next, we investigated how YAP-mediated exosome secretion-promoting transcription system regulated transformation phenotypes of LUAD cells. We observed that YAP could induce cell viability, colony formation while reduce caspase 3/7 activity, and these effects were further enhanced by SOX10 and Mycn overexpression and reversed by knockout of NSUN2 and ALYREF. Similarly, NSUN2 and ALYREF could further enhance YAP-mediated increasing cell viability, colony formation and decreasing caspase 3/7 activity, whereas these effects could be reversed by an exosome inhibitor GW4869 treatment (Fig. 6A–D, Fig. S6A–D). We used CCK8 experiments to measure the cell viability in control, YAP overexpression, YAP, SOX10 and Mycn overexpression, as well as YAP, NSUN2 and ALYREF overexpression cells, and added GW4869 to each group to eliminate the influence of exosome. Interestingly, we found that there was no statistically significant difference in cell viability among the four groups (Fig. S6E), which excluded the influence of other factors except for exosome on cell proliferative capacity. Xenograft experiments revealed that YAP co-expressed with Mycn and SOX10 significantly increased tumor volume, whereas this effect could be reversed by NSUN2 and ALYREF knockout; also, YAP co-expressed with NSUN2 and ALYREF significantly raised tumor volume, and this effect could be reversed by GW4869 treatment (Fig. 6E–H). Next, we established two series of LUAD patient derived xenograft (PDX) models, and the morphology of the tissue was verified by H&E staining. (Fig. S6F). PDX1 highly expressed YAP and NSUN2, while PDX2 lowly expressed YAP and NSUN2 (Fig. 6I–K). Compared to PDX2, the exosome concentration and YAP m^5^C level were much higher in PDX1 (Fig. S6G, H), and the transplanted tumor size in PDX1 was significantly larger (Fig. 6L). These data suggested YAP m^5^C modification and its downstream exosome secretion-promoting transcription system are both important for transformation phenotypes of LUAD.

### YAP m^5^C modification and downstream exosome secretion-promoting transcription system lead to AZD9291 resistance

**Fig. 7:**
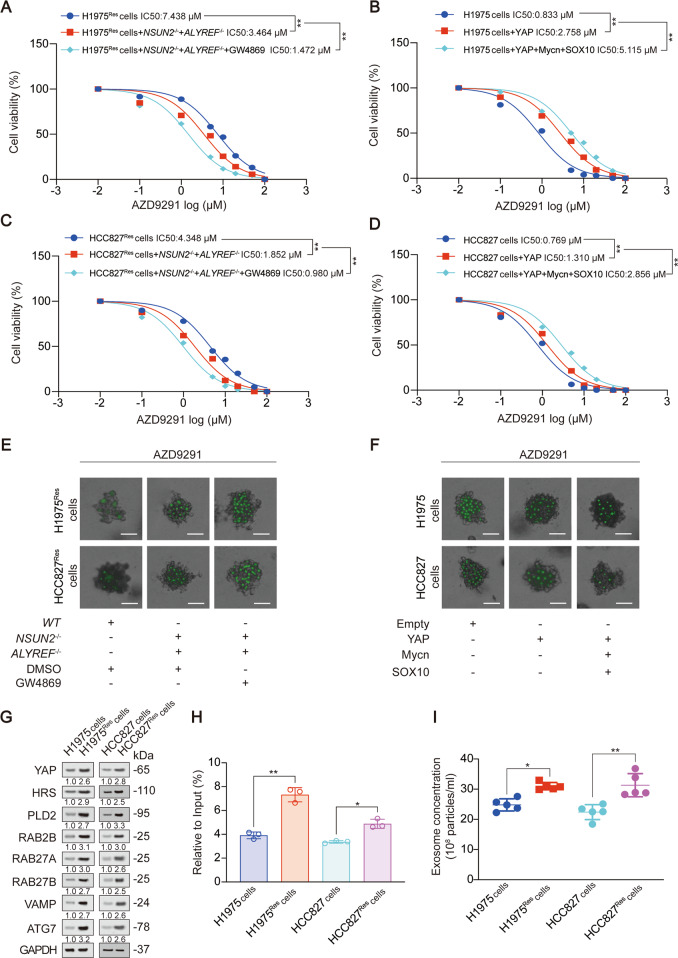
YAP m^5^C modification and downstream exosome secretion-promoting transcription system causes AZD9291 resistance. **A**–**D** IC50 was calculated in AZD9291 (24 h) treated H1975 AZD9291 resistant (H1975^Res^, **A**), normal H1975 (**B**), HCC827^Res^ (**C**), normal HCC827 (**D**) cells with indicated treatment. GW4869 (10 µM, 24 h). **E** 3D spheroids were generated by NSUN2 with or without ALYREF knocked out H1975^Res^ and HCC827^Res^ cells. After the formation of 3D spheroids, AZD9291 (10 µM, 24 h) with or without GW4869 (10 µM, 24 h) was treated. Then cells were stained with SYTOX green. Representative images and graphed data are shown, Scale bar, 50 µm. **F** 3D spheroids were generated by YAP with or without Mycn and SOX10 overexpressed H1975 and HCC827 cells. After the formation of 3D spheroids, AZD9291 (10 µM, 24 h) was treated. Then cells were stained with SYTOX green. Representative images and graphed data are shown, Scale bar, 50 µm. **G**–**I** Indicated protein levels (**G**), YAP m^5^C level (**H**), exosome concentration (**I**) were measured in H1975, H1975^Res^, HCC827 and HCC827^Res^ cells. The level of proteins was normalized to that of GAPDH, and the normalized level of proteins in negative control groups were arbitrarily set to 1. The data are shown as the means ± SD from 3 to 5 independent experiments. Images of IB are representative ones of three independent experiments. ^*^p < 0.05, ^**^p < 0.01 indicate statistical significance. The data from (**A**–**D**) were analyzed by a two-way ANOVA test. The data from (**H**, **I**) were analyzed by a student’s *t* test.

We also explored whether YAP-mediated exosome secretion-promoting transcription system would cause resistance of third generations of EGFR-TKI. We used AZD9291-resistant H1975 and HCC827 cell lines from previous studies to investigate this question [5]. Through IC50 value determination and 3D cell culture, we observed that knockout of NSUN2 and ALYREF significantly inhibited the AZD9291 resistance of LUAD cells, and GW4869 treatment further aggravated this phenomenon. On the contrary, the overexpression of YAP stimulated the resistance of LUAD cells to AZD9291, while the overexpression of Mycn and SOX10 further promoted this phenomenon (Fig. 7A–F, Fig. S7A–B). We also found that protein levels of YAP and its related exosome secreting genes, YAP m^5^C modification level and exosome concentration in AZD9291 drug-resistant cells were significantly higher than those in non-drug-resistant LUAD cells (Fig. 7G–I). Therefore, we suggested that YAP-mediated exosome secretion-promoting transcription system would lead to resistance of third generations of EGFR-TKI in LUAD.

### Exosome autocrine has no additional effect for LUAD cell viability and resistance to AZD9291

In order to evaluate the autocrine effect of the exosome, we first used cell culture medium with the same treatment to replace the original medium at 4, 8 and 16 h after finishing cell transfection (24 h after transfection) to weaken the autocrine effect of the exosome. However, we found that such treatment did not affect cell viability in the control group, YAP overexpression group, YAP, SOX10 and Mycn overexpression group, as well as YAP, SOX10 and Mycn overexpression combined with NSUN2 and ALYREF knockout group (Fig. S7C). Subsequently, we used cell culture medium with the same treatment to replace the original medium at 4, 8 and 16 h after AZD9291 treatment to weaken the autocrine effect of the exosome when we measure the IC50 in the control group, YAP overexpression group, YAP, SOX10 and Mycn overexpression group, as well as YAP, SOX10 and Mycn overexpression combined with NSUN2 and ALYREF knockout group. Similarly, we observed that the IC50 value of AZD9291 treated cells was not affected by the change of culture medium (Fig. S7D). These data suggested that exosome autocrine did not additionally affect LUAD cell viability and resistance to AZD9291.

## Discussion

As an important signal node in cells, YAP can control organ size and promote tumor development by regulating transcription [44]. Therefore, it is possible to find a more effective way to inhibit YAP by analyzing its upstream regulation mechanism. In addition to Hippo phosphorylation kinase cascade [11], as well as ERBB2, CREB, and HBP [7, 45, 46], YAP is also regulated by Wnt, PI3K/AKT/mTOR, GPCR signaling, SFKs and VGLL4 [47–49]. These findings confirm that the mechanisms by which YAP is regulated are very broad. RNA modifications also regulate YAP by affecting the stability of YAP mRNA. For instance, FTO depletion increased YAP m^6^A modification at mRNA 3′ UTR, accelerating the degradation of YAP mRNA [50]. Our Lab also confirmed that m^6^A modification of YAP 3′ UTR leads to the degradation of YAP mRNA [31]. Here, we found a m^5^C modification motif near the m^6^A modification motif. The increase of m^5^C modification led to the decrease of m^6^A modification and reduced the binding of miR-582-3p to YAP 3′ UTR. It’s possible that in the 3′ UTR region of YAP, there is a dynamic balance of m^5^C and m^6^A modification, thereby regulating the stability of YAP mRNA.

In this study, we divided the 3′ UTR of YAP into five parts to explore its m^5^C modification. First of all, when exploring the m^5^C modification of YAP 3′UTR, it must be divided to investigate, otherwise it would be difficult to find the exact m^5^C site, and the long product would reduce the amplification efficiency of qPCR [51]. As for why the 3′UTR of YAP was further subdivided, the first reason is that the complexity of the experiment would be increased; the second reason is that there are more m^5^C sites besides our findings, and too much subdivision would reduce the statistical significance among each segment; and the third reason is that five divided segments were sufficient for subsequent exploration. Therefore, there was no further subdivision in our research. Of course, it could be seen from the results that the m^5^C-modified region of YAP 3′UTR is by no means limited to the 328–331 motif we found, and there might be different situations in different cell lines. In follow-up studies, researchers can further develop a more comprehensive discussion of YAP m^5^C modifications by exploring the status of m^5^C modifications in other YAP 3′UTR regions, as well as m^5^C modifications occurring in other YAP mRNA regions.

Recently, researchers found that YAP can promote cancer by regulating the tumor microenvironment [1]. For example, YAP stimulates the transcription of PDL1, and YAP-induced PDL1 expression protects tumor cells from T cell cytotoxicity, and treatment with PD1 inhibitors restores T cell effector functions, resulting to the killing of YAP-expressing tumor cells [52]. In addition, YAP is upregulated in Treg cells to enforce their capacity to suppress antitumor immunity [53]. Exosomes are master cellular regulators in tumor microenvironment and critical messengers that participate in cell-to-cell communications [54]. There are some reports on the regulation of YAP by components in tumor exosomes. For example, Wnt5a, Wnt5b, long non-coding ASMTL-AS1 in tumor cell exosomes all have the ability to activate YAP to promote tumor formation and development [55, 56]. However, studies of YAP regulating exosome generation are rare. In addition, no studies have shown that the 3′ UTR of YAP is related to the secretion of exosomes at present. Our study demonstrated that the 3′ UTR of YAP is modified by m^5^C, and this modification depends on NSUN2 and ALYREF. We made it clear that NSUN2 and ALYREF could promote exosome secretion only in the presence of YAP. Therefore, these data innovatively demonstrated that YAP 3′ UTR could play a role in promoting exosome secretion. Subsequently, by screening a series of exosome biogenesis, transport and release genes, we found that YAP simultaneously promoted the transcription of HRS, PLD2, RAB2B, RAB27A, RAB27B, VAMP, ATG7, and the transcription factors that played a direct role in promoting transcription were Mycn and SOX10, which were the first time found to act as YAP-related transcription factors.

High expression of YAP leads to resistance to various types chemotherapies, immunotherapies and targeted therapies. For example, taxane is a major class of anti-microtubule chemotherapy drugs widely used in treatment of ovarian, lung, breast, cervical and prostate cancer and acts to stabilize GDP-bound tubulin, thereby preventing microtubule disassembly during cell cycle [57]. miRNA-363, which is upregulated in taxane-resistant cancer cells, confers resistance by degrading LATS mRNA, which in turn dephosphorylates and activates YAP [58]. The transcriptional activation of multiple downstream genes of YAP can also lead to chemoresistance. For example, YAP activation upregulates PD-L1 transcription and leads to immunotherapy resistance [52], and is a major resistance mechanism to EGFR inhibitors [19]. Our study is the first to clarify that YAP is associated with resistance to third-generation EGFR-TKIs, and this resistance may be related to the YAP m^5^C modification and the its function of promoting exosome secretion. It has been reported that exosomes can lead to resistance of third-generation EGFR-TKI [59, 60], and other key downstream genes of YAP promoting resistance of third-generation EGFR-TKI remain to be explored.

**Fig. 8:**
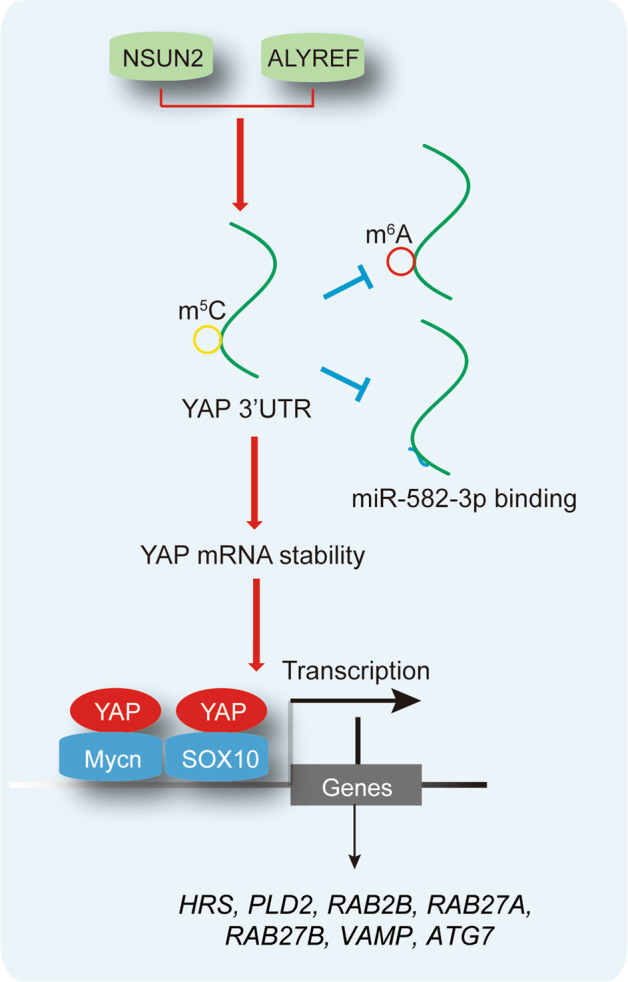
Schematic representation of the study. Briefly, in LUAD, YAP m^5^C modification on its 3′UTR depends on NSUN2 and ALYREF, and this m^5^C modification inhibits its m^6^A modification and miR-582-3p binding. Furthermore, YAP m^5^C modification increases YAP mRNA stability and stimulates Mycn- and SOX10-dependent exosome secretion-related genes including *HRS*, *PLD2*, *RAB2B*, *RAB27A*, *RAB27B*, *VAMP* and *ATG7*.

In conclusion, YAP m^5^C modification increases its mRNA stability and promotes the Mycn- and SOX10-dependent transcription of exosome secretion-related genes in LUAD (Fig. 8). Targeting YAP m^5^C modification will be helpful for future LUAD treatment.

## Supplementary information

Supplementary figures

Supplementary table

## Data Availability

All data generated or analyzed during this study are included in this paper and its supplementary files.
