# Supplementary material for: YAP 5-methylcytosine modification increases its mRNA stability and promotes the transcription of exosome secretion-related genes in lung adenocarcinoma
Source: Cancer Gene Ther. 2022 Sep 19;30(1):149–62. doi: 10.1038/s41417-022-00533-7 (PMC9842506; doi:10.1038/s41417-022-00533-7)
Supplement: Supplementary file 1 — Supplementary figures [file 41417_2022_533_MOESM1_ESM.docx]

**Figure S1, supplemented to Figure 1.**


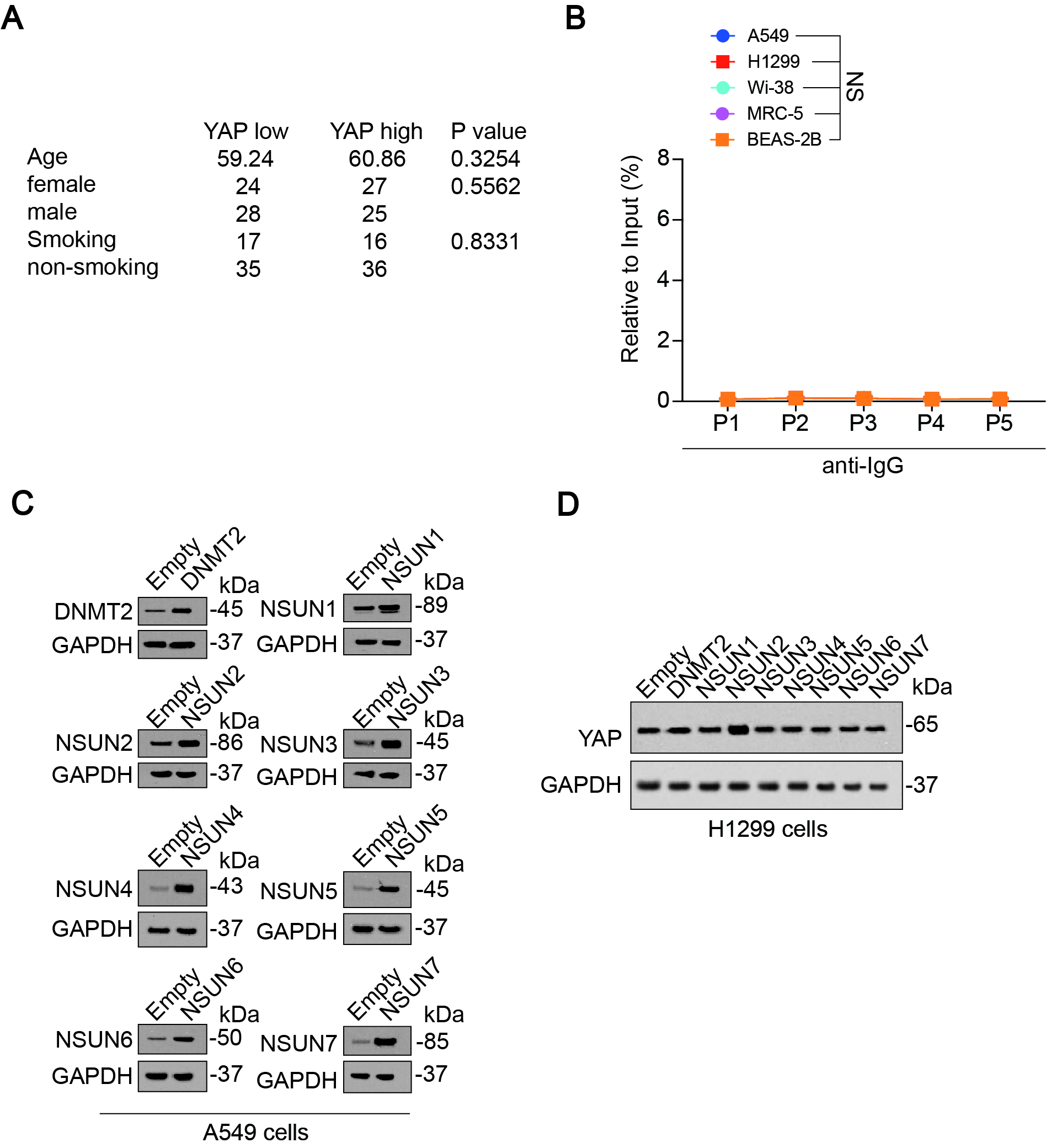


**Figure S1, supplemented to Figure 1.**

(A) Correlation between YAP and age, gender or smoking habit in LUAD patients.

(B) The parallel experiments for Fig. 1E using anti-IgG.

(C) IB experiments proved the efficiency of m^5^C writer overexpression plasmids in A549 cells.

(D) YAP protein level was measured by IB in H1299 cells with indicated plasmids overexpressed.

The data are shown as the means ± SD from 3 independent experiments. Images of IB are representative ones of 3 independent experiments. **, p < 0.01 indicates statistical significance. The data from Panel A were analyzed by a student’s t test and a χ^2^ test. The data from Panel B were analyzed by a one-way and two-way ANOVA test.

**Figure S2, supplemented to Figure 2.**


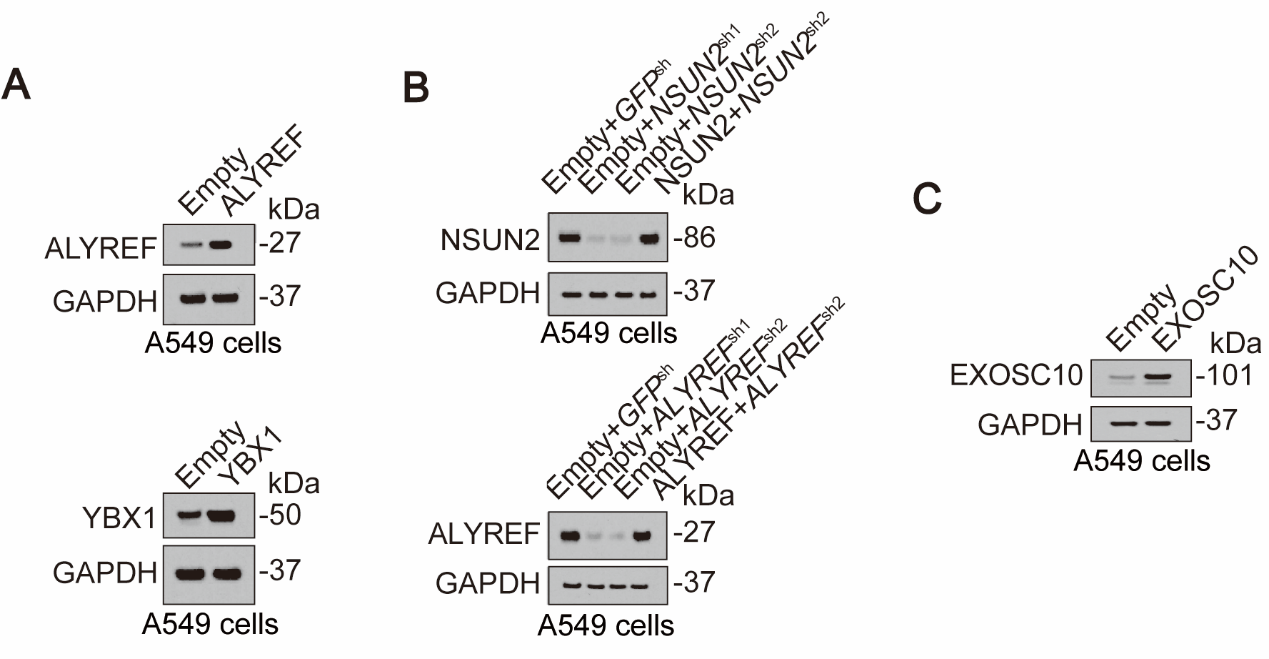


**Figure S2, supplemented to Figure 2.**

(A) IB experiments proved the efficiency of ALYREF and YBX1 overexpression plasmids in A549 cells.

(B) IB experiments proved the efficiency of NSUN2 and ALYREF knockdown plasmids in A549 cells.

(C) IB experiments proved the efficiency of EXOSC10 overexpression plasmids in A549 cells.

Images of IB are representative ones of 3 independent experiments.

**Figure S3, supplemented to Figure 3.**

**
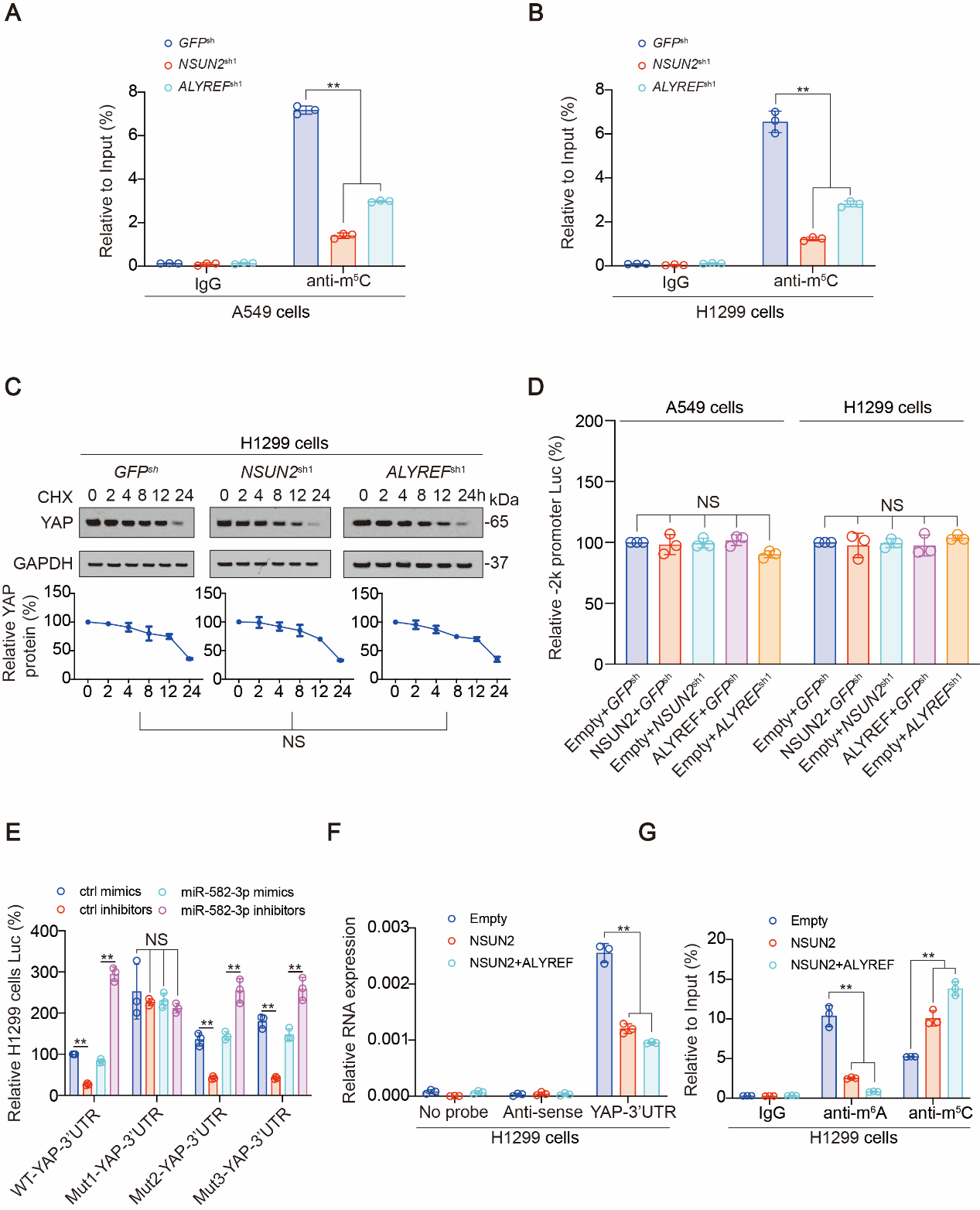
**

**Figure S3, supplemented to Figure 3.**

(A-B) The enrichment of m^5^C in A549 (A) and H1299 (B) cells at P1 region of YAP 3′UTR was calculated as the percentage of the input RNA via RNA-IP using the anti-m^5^C antibodies.

(C) YAP protein stability was measured in H1299 cells with NSUN2 or ALYREF knockdown at indicated time after CHX treatment.

(D) YAP -2k promoter activity was measured in A549 and H1299 cells with NUSN2 or ALYREF overexpressed or knocked down.

(E) Activities of WT-YAP-3′UTR, Mut1-YAP-3′UTR, Mut2-YAP-3′UTR, or Mut3-YAP-3′UTR were measured in H1299 cells with miR-582-3p overexpressed or knocked down.

(F) miR-582-3p in NSUN2 with or without ALYREF overexpressed H1299 cell lysis was pulled down and enriched with a YAP 3’UTR P1 region probe and then detected by qPCR.

(G) The enrichment of m^6^A and m^5^C in NSUN2 with or without ALYREF overexpressed H1299 cells at P1 region of YAP 3′UTR was calculated as the percentage of the input RNA via RNA-IP using the anti-m^6^A and anti-m^5^C antibodies.

The data are shown as the means ± SD from 3 independent experiments. Images of IB are representative ones of 3 independent experiments. **, p < 0.01 indicates statistical significance. NS, non-significant. The data from Panel A-B, D-G were analyzed by a one-way ANOVA test. The data from Panel C were analyzed by a two-way ANOVA test.

**Figure S4, supplemented to Figure 4.**

**
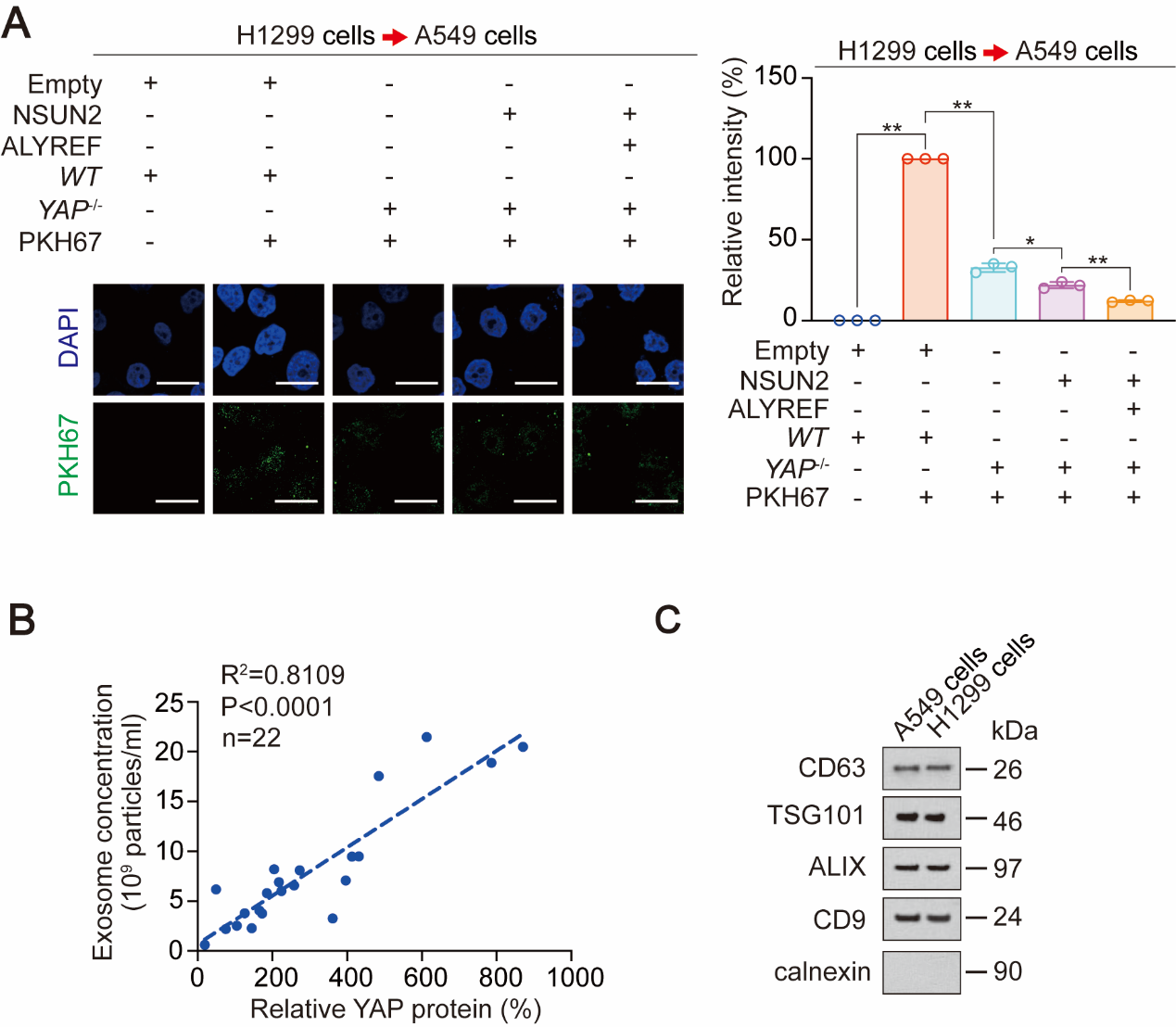
**

**Figure S4, supplemented to Figure 4.**

(A) The exosome from H1299 cells with YAP knocked out, with or without NSUN2 and ALYREF overexpression was marked by PKH67 (green) and incubated with A549 cells. Afterwards, IF was performed for the detection of exosome. Scale bar, 50 µm.

(B) Correlation between plasma exosome concentration and tissue YAP protein level in LUAD samples.

(C) Representative IB images of exosome biomarkers (CD63, TSG101, ALIX and CD9) and ER biomarker Calnexin in exosome from A549 and H1299 cells.

The data are shown as the means ± SD from 3 independent experiments. Images of IB are representative ones of 3 independent experiments. The data from Panel A were analyzed by a one-way ANOVA test. The data from Panel B were analyzed by Spearman rank-correlation analysis.

**Figure S5,** **supplemented to Figure 5.**

**
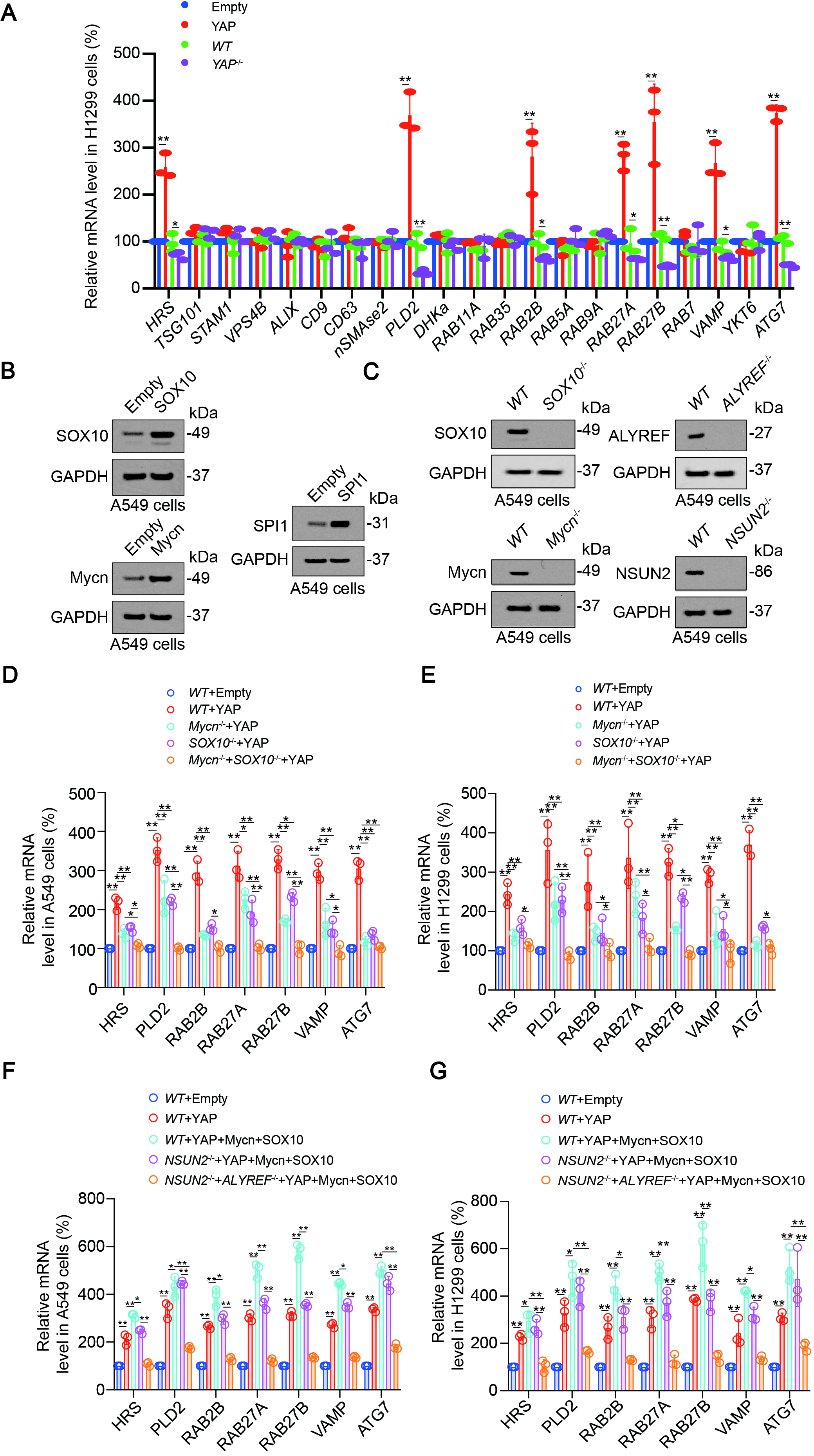
**

**Figure S5,** **supplemented to Figure 5.**

(A) Indicated mRNA levels were measured by qPCR in YAP overexpressed or knocked out H1299 cells.

(B) IB experiments proved the efficiency of Mycn, SOX10 and SPI1 overexpression plasmids in A549 cells.

(C) IB experiments proved the efficiency of SOX10, Mycn, ALYREF and NSUN2 knockout plasmids in A549 cells,

(D-E) HRS, PLD2, RAB2B, RAB27A, RAB27B, VAMP and ATG7 mRNA levels in A549 (D) andH1299 (E) cells with YAP overexpressed with or without Mycn or SOX10 knockout.

(F-G) HRS, PLD2, RAB2B, RAB27A, RAB27B, VAMP and ATG7 mRNA levels in A549 (F) and H1299 (G) cells with indicated genes overexpressed or knocked out.

The data are shown as the means ± SD from 3 independent experiments. Images of IB are representative ones of 3 independent experiments. *, p < 0.05, **, p < 0.01 indicate statistical significance. The data from Panel A were analyzed by a student’s t test. The data from Panel D-G were analyzed by a one-way ANOVA test.

**Figure S6,** **supplemented to Figure 6.**


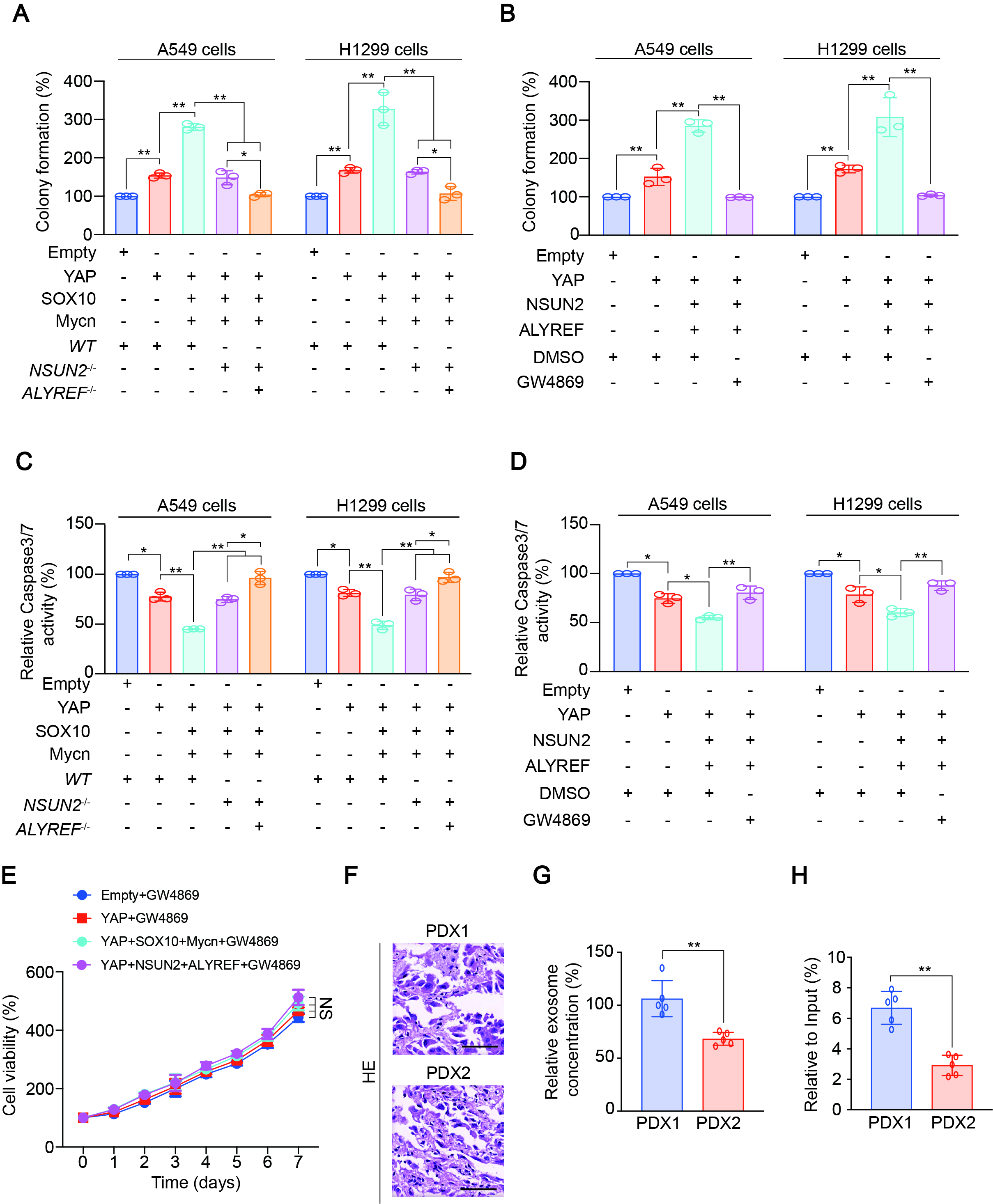


**Figure S6,** **supplemented to Figure 6.**

(A-B) Colony formation was measured in A549 or H1299 cells with indicated genes overexpressed or knocked out.

(C-D) Relative Caspase 3/7 activity was measured in A549 or H1299 cells with indicated genes overexpressed or knocked out.

(E) Cell viability was measured at the indicated time in control, YAP overexpressed, YAP, SOX10, Mycn overexpressed, and YAP, NSUN2, ALYREF overexpressed A549 cells with GW4869 (10 µM) treatment.

(F) Representative H&E staining PDX1 and PDX2 tissues, scale bar, 100 µm.

(G-H) Exosome concentration (G) and YAP m^5^C level (H) in PDX1 and PDX2 tissues.

The data are shown as the means ± SD from 3-5 independent experiments. *, p < 0.05, **, p < 0.01 indicate statistical significance. The data from Panel A-D were analyzed by a one-way ANOVA test. The data from Panel E were analyzed by a two-way ANOVA test. The data from Panel G-H were analyzed by a student’s t test.

**Figure S7, supplemented to Figure 7.**


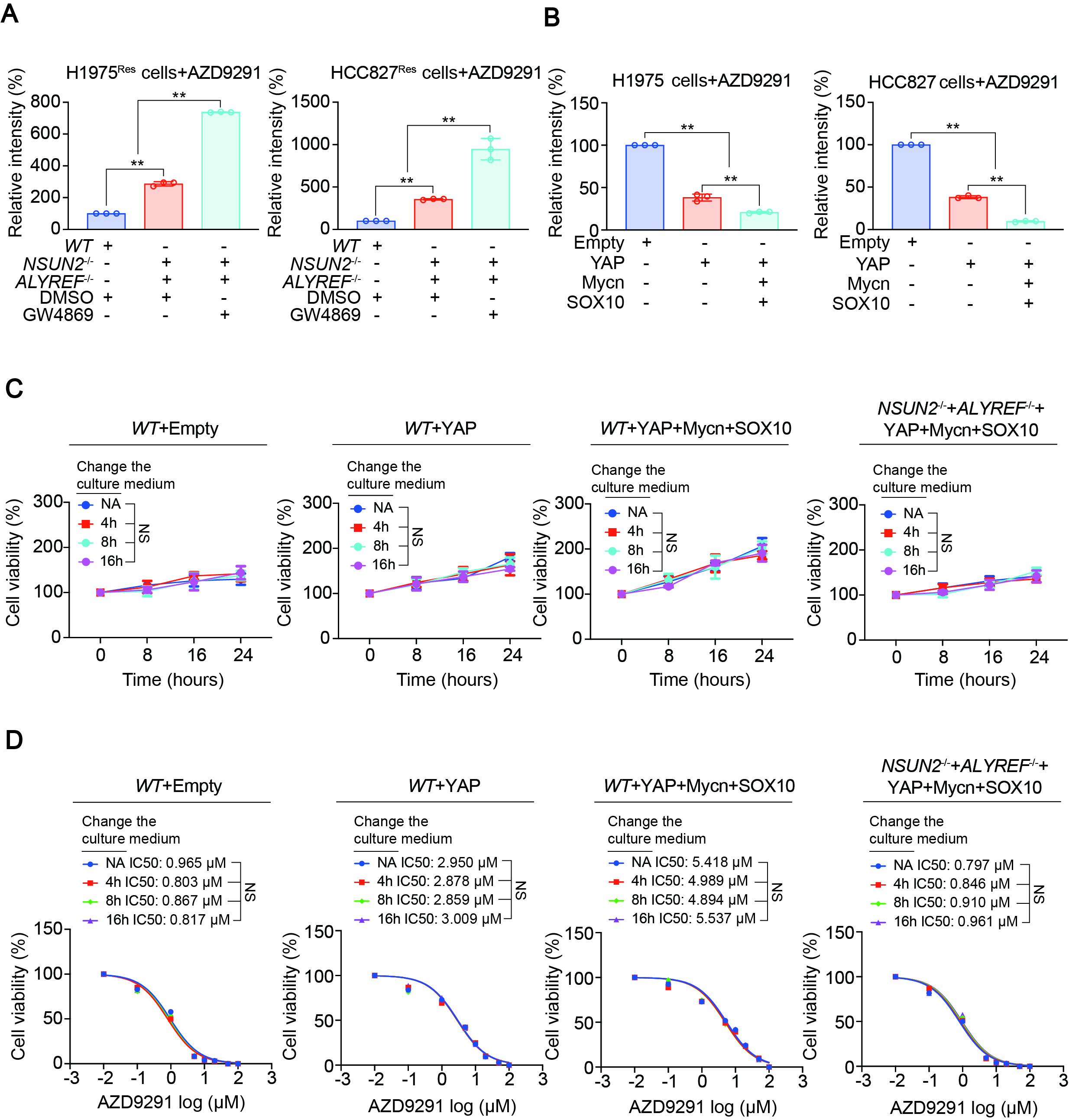


**Figure S7, supplemented to Figure 7.**

(A-B) Relative intensity of SYTOX green staining for 3D spheroids culture related to Figure 7E (A) and 7F (B).

(C) Cell viability was measured in control, YAP overexpressed, YAP, Mycn, SOX10 overexpressed, as well as YAP, Mycn, SOX10 overexpressed combined with NSUN2 and ALYREF knocked out A549 cells at 0, 8, 16, 24 hours after finishing cell transfection (24 hours after transfection). The original medium was replaced with the culture medium with the same treatment at 4, 8 and 16 hours as indicated.

(D) IC50 was calculated in AZD9291 (24 hours) treated control, YAP overexpressed, YAP, Mycn, SOX10 overexpressed, as well as YAP, Mycn, SOX10 overexpressed combined with NSUN2 and ALYREF knocked out normal H1975 cells. The original medium was replaced with the culture medium with the same treatment at 4, 8 and 16 hours as indicated.

The data in Panel A-B were analyzed by a one-way ANOVA test. The data in Panel C-D were analyzed by a two-way ANOVA test. **, p < 0.01 indicates statistical significance.

**Figure S8.**

**Figure S8. Represented images of original blots.**

(A1, A2) Original blots for Fig. 1H.

(B1-B4) Original blots for Fig. 2C and Fig. 2D.

(C1-C6) Original blots for Fig. 3A.

(D1-D14) Original blots for Fig. 3C.

(E1-E8) Original blots for Fig. 5G.

(F1-F8) Original blots for Fig. 5I.

(G1-G6) Original blots for Fig. 6G.

(H1-H9, I1-I9) Original blots for Fig. 7G.

(J1-J16) Original blots for Fig. S1C.

(K1-K2) Original blots for Fig. S1D.

(L1-L4) Original blots for Fig. S2A.

(M1-M4) Original blots for Fig. S2B.

(N1-N2) Original blots for Fig. S2C.

(O1-O6) Original blots for Fig. S3C.

(P1-P5) Original blots for Fig. S4C.

(Q1-Q6) Original blots for Fig. S5B.

(R1-R8) Original blots for Fig. S5C.
