# Supplementary material for: YAP 5-methylcytosine modification increases its mRNA stability and promotes the transcription of exosome secretion-related genes in lung adenocarcinoma
Source: Cancer Gene Ther. 2022 Sep 19;30(1):149–62. doi: 10.1038/s41417-022-00533-7 (PMC9842506; doi:10.1038/s41417-022-00533-7)
Supplement: Supplementary file 2 — Supplementary table [file 41417_2022_533_MOESM2_ESM.docx]

**Table S1.** Primers, probes, shRNAs and sgRNAs in the study.

| YAP-qPCR-F1 | CAGGCAGACTGAATTCTAAATC |
| --- | --- |
| YAP-qPCR-R1 | AATGCACACCCACAAAATTATCC |
| YAP-qPCR-F2 | GGGTTGTTCTGTTTTGTTGGG |
| YAP-qPCR-R2 | ACAAATGAGAACTACAAACTAGGGG |
| YAP-qPCR-F3 | GGAATGAACACATTAACGACTAG |
| YAP-qPCR-R3 | TCAAGATTTAATCTTTCTGCTGGGG |
| YAP-qPCR-F4 | ATTAAGTCTGGGGGGAAATGGCCACTG |
| YAP-qPCR-R4 | GGTTTATTGTAAAAGCAATTCGGG |
| YAP-qPCR-F5 | GGGGTTATCAACTTTTTACATTTGTG |
| YAP-qPCR-R5 | GAGTTTAAGGAAAGAATGGG |
| GAPDH-qPCR-F | ATCATCCCTGCCTCTACTGG |
| GAPDH-qPCR-R | GTCAGGTCCACCACTGACAC |
| miR-582-3p-qPCR-F | GCCTATAACTGGTTGAACAAC |
| miR-582-3p-qPCR-R | GCAGGGTCCGAGGTATTC |
| miR-582-3p-RT-qPCR | GTCGTATCCAGTGCAGGGTC CGAGGTATTCGCACTGGATACGACGGTTCA |
| u6-qPCR-F | CTCGCTTCGGCAGCACA |
| u6-qPCR-R | AACGCTTCACGAATTTGCGT |
| YAP-3'UTR-probe-1 | GATTGGTGAACATTTAACTGGGCAAAAGGAA |
| YAP-3'UTR-antisense-1 | TTCCTTTTGCCCAGTTAAATGTTCACCAATC |
| HRS-qPCR-F | AGTGGCTGTCGGGTATTCATC |
| HRS-qPCR-R | CCGTCCATATCCCTTGAAGAATC |
| TSG101-qPCR-F | GAGAGCCAGCTCAAGAAAATGG |
| TSG101-qPCR-R | TGAGGTTCATTAGTTCCCTGGA |
| STAM1-qPCR-F | AATCCCTTCGATCAGGATGTTGA |
| STAM1-qPCR-R | CGAGACTGACCAACTTTATCACA |
| VPS4B-qPCR-F | ATGTCATCCACTTCGCCCAAC |
| VPS4B-qPCR-R | TTGCTTGGCTTTATCACCCTG |
| CD9-qPCR-F | TCCACTATGCGTTGAACTGCT |
| CD9-qPCR-R | GGTTTCGAGTACGTCCTTCTTG |
| CD63-qPCR-F | ATGCAGGCAGATTTTAAGTGCT |
| CD63-qPCR-R | GTTCTTCGACATGGAAGGGATTT |
| nSMase2-qPCR-F | GCTGCCCTTTGCGTTTCTC |
| nSMase2-qPCR-R | TCCAGCCGTGAATAGATGTAGG |
| PLD2-qPCR-F | CAGATGGAGTCCGATGAGGTG |
| PLD2-qPCR-R | CCGCTGGTATATCTTTCGGTG |
| RAB11A-qPCR-F | CAACAAGAAGCATCCAGGTTGA |
| RAB11A-qPCR-R | GCACCTACAGCTCCACGATAAT |
| RAB35-qPCR-F | TACTGTTGCGTTTTGCAGACA |
| RAB35-qPCR-R | CCCCGATAATACGTGGAGGTG |
| RAB2B-qPCR-F | GTACGACATTACAAGGCGTGA |
| RAB2B-qPCR-R | ATGTTGGAACTAGAGTGCTGC |
| RAB5A-qPCR-F | CAAGGCCGACCTAGCAAATAA |
| RAB5A-qPCR-R | GATGTTTTAGCGGATGTCTCCAT |
| RAB9A-qPCR-F | AGGGACAACGGCGACTATC |
| RAB9A-qPCR-R | TCTGACCTATCCTCGGTAGCA |
| RAB27A-qPCR-F | GCTTTGGGAGACTCTGGTGTA |
| RAB27A-qPCR-R | TCAATGCCCACTGTTGTGATAAA |
| RAB27B-qPCR-F | TAGACTTTCGGGAAAAACGTGTG |
| RAB27B-qPCR-R | AGAAGCTCTGTTGACTGGTGA |
| RAB7-qPCR-F | GTGTTGCTGAAGGTTATCATCCT |
| RAB7-qPCR-R | GCTCCTATTGTGGCTTTGTACTG |
| YKT6-qPCR-F | TGGTCACCTCAGTAGATACCAG |
| YKT6-qPCR-R | CTCGCTCTAACAGAGACTCCA |
| ALIX-qPCR-F | ATCGCTGCTAAACATTACCAGTT |
| ALIX-qPCR-R | AGGGTCCCAACAGTATCTGGA |
| ATG7-qPCR-F | ATGATCCCTGTAACTTAGCCCA |
| ATG7-qPCR-R | CACGGAAGCAAACAACTTCAAC |
| DGKa-qPCR-F | GGTGTGTCTCAATGATGTTTCCT |
| DGKa-qPCR-R | GTCCGTGTCGTACAGCTTGAA |
| VAMP-qPCR-F | GAGGTTCCAGACTACTTACGGT |
| VAMP-qPCR-R | GACACTTGAGAACTCGCTATTCA |
| NSUN2-sh1 | GCTACATGCCGACATGAATGT |
| NSUN2-sh2 | GCGTGTTAGAAATCACTTGTT |
| ALYREF-sh1 | CGTGGAGACAGGTGGGAAACT |
| ALYREF-sh2 | GCAAATCCGCGTGCGGAACAG |
| NSUN2-sg | GCGCCATCCTCCGCGTCCTC |
| ALYREF-sg | GACGCCGTTGTACTGCTTCA |
| Mycn-sg | GTGCTCCGCGAAGCCACGGC |
| SOX10-sg | GCTGGCGCCGTTGACGCGCA |
